# Slow Magnetic Relaxation in a Rare, Neutral, Formally Divalent Terbium Bis(amide) Complex

**DOI:** 10.1021/acs.inorgchem.4c05349

**Published:** 2025-05-20

**Authors:** Florian Benner, Elizabeth R. Pugliese, Ernesto Castellanos, Saroshan Deshapriya, Selvan Demir

**Affiliations:** Department of Chemistry, 3078 Michigan State University, 578 South Shaw Lane, East Lansing, Michigan 48824, United States

## Abstract

Neutral organometallic complexes containing a formally divalent terbium ion are especially scarce, owing to the highly negative Tb^III^/Tb^II^ reduction potential and the thermodynamic stability of the Tb^III^ ion. In fact, there are only two crystallographically characterized neutral terbium­(II) complexes known to date. Here, we present the synthesis of the unprecedented heteroleptic Tb­(III) bis­(amide) chloride complex, (NHAr*)_2_TbCl (**1**) (where Ar* = 2,6-(Ar′)_2_C_6_H_3_, Ar′ = 2,4,6-(^*i*^Pr)_3_C_6_H_2_), which was chemically reduced using the strong reducing agent, KC_8_, to yield a new member of this small family of highly reactive Tb^II^ compounds, namely, the homoleptic terbium bis­(amide) complex, (NHAr*)_2_Tb (**2**). Notably, the spectroscopic and magnetic characterization of **2** revealed that the compound contains a formally divalent terbium ion since the additional electron was exclusively found to reside in the primarily arene ligand-based π* orbitals, as corroborated by *ab initio* calculations. Furthermore, **2** exhibits slow magnetic relaxation between 1.8 and 16 K under a 1250 Oe applied dc field. *Ab initio* calculations uncovered that magnetic relaxation occurs through the first excited spin–orbit state. Study of **2** via SQUID magnetometry hints at considerably weaker 4f–5d magnetic coupling relative to other Tb^II^ complexes, rendering its electronic structure unique relative to that of other neutral organometallic Tb^II^ complexes.

## Graphical abstract

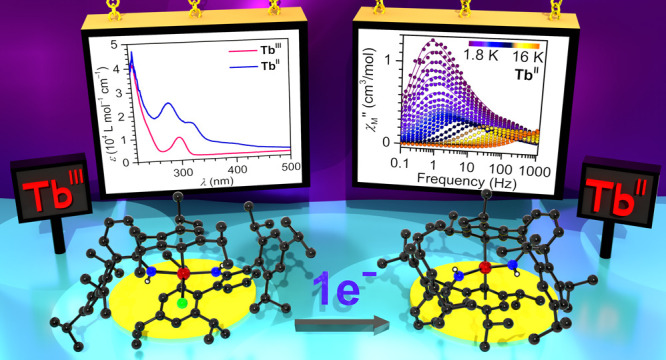

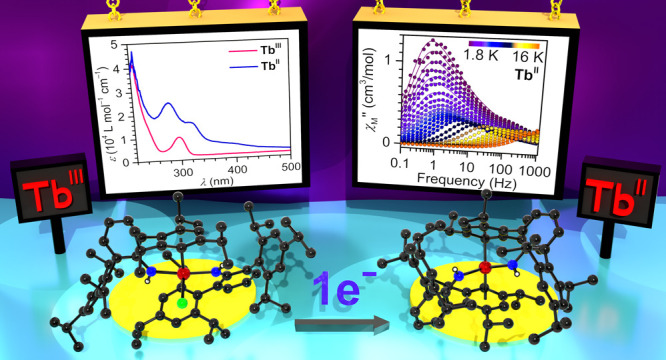

## Introduction

While the most stable oxidation state of the lanthanides (Ln) is +3, divalent Ln ions could be successfully stabilized through designing judiciously tailored coordination environments for all 4f-elements, except for radioactive Pm. The classical Ln^II^ ions benefit from favorable electron configurations (Sm^II^, 4f^6^; Eu^II^, 4f^7^; Yb^II^, 4f^14^), rendering Ln^II^ complexes of these metals readily accessible.^1^ However, Tb^II^ complexes remain exceedingly scarce due to its highly reactive and strongly reducing nature, as evidenced by the very low Tb^III^/Tb^II^ redox potential of −2.95 V.^2–4^ Despite the synthetic rigor required to synthesize reduced lanthanide complexes, the divalent oxidation state has proven critical in the advancement of catalysis,^5,6^ small-molecule activation,^7^ quantum information technologies,^8^ and single-molecule magnetism.^9,10^ Terbium in particular is interesting in its divalent oxidation state (4f^8^5d^1^) since it is a Kramers ion with an inherently bistable ground state with large orbital angular momentum, which altogether engenders large magnetic anisotropy, rendering it an ideal candidate for single-molecule magnet (SMM) design. Notably, despite its non-Kramers nature, Tb­(III) can also give remarkable multinuclear SMMs when radicals are used as an exchange bias.^11–13^

The spin-based properties of the Tb^II^ ion stem from its unique electronic structure. For the trivalent Tb^III^ ion, all valence electrons reside within the deeply contracted 4f shell, which hinders effective metal–ligand orbital overlap. By contrast, the additional electron in divalent Tb complexes can be potentially housed in various orbitals, spanning from 5d, 6s, and 6p to even ligand orbitals, greatly impacting the magnetic properties of these types of complexes.

To date, only a few stable, yet isolable Tb^II^ complexes are known,^3,10,14–19^ where a small fraction are neutral molecules (Figure S1), featuring a homoatomic first coordination sphere: (A) carbon atoms in the bis­(cyclopentadienide) complex, (Cp^*i*Pr5^)_2_Tb,^10^ and (B) nitrogen atoms in the bis­(amidinate) complex, (Piso)_2_Tb^14^ (Figure 1). Neutral, divalent Tb complexes with a heteroatomic first coordination sphere remain elusive. Herein, the synthesis and characterization of two terbium bis­(amide) complexes, heteroleptic (NHAr*)_2_TbCl, **1**, and homoleptic (NHAr*)_2_Tb, **2** (where Ar* = 2,6-(Ar′)_2_C_6_H_3_, Ar′ = 2,4,6-(^*i*^Pr)_3_C_6_H_2_), are reported. Recently, this ligand scaffold was utilized to produce remarkable dysprosium single-molecule magnets.^20^

**Figure 1:**
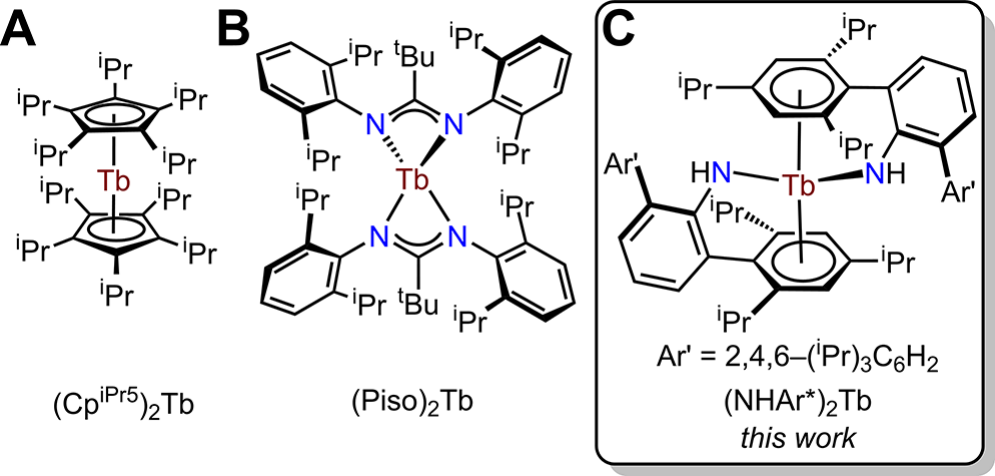
All known and crystallographically characterized neutral, divalent terbium complexes: (Cp^*i*Pr5^)_2_Tb (A), (Piso)_2_Tb (B), and (NHAr*)_2_Tb (C). Abbreviations: Cp^*i*Pr5^ = C_5_^*i*^Pr_5_; Piso = {N­(2,6-(^*i*^Pr)_2_C_6_H_3_)}_2_C^*t*^Bu; Ar* = 2,6-(Ar′)_2_C_6_H_3_, Ar′ = 2,4,6-(^*i*^Pr)_3_C_6_H_2_.

Single-crystal X-ray diffraction analysis revealed that the terbium ions in the bis­(terphenylamide) complexes exhibit ligation to both nitrogen atoms and metal–arene π-interactions. Notably, **2** joins a small set of formally divalent Tb complexes. Subjecting **2** to a dc field uncovered slow magnetic relaxation, rendering **2** as the third known single-molecule magnet innate to a Tb^II^ center. Furthermore, subjecting **2** to SQUID magnetometry concomitant with performing *ab initio* calculations revealed an electronic structure distinctly different from hitherto known Tb^II^ complexes. A strong Tb 5d and ligand π* orbital hybridization is identified as the detrimental factor for a faster magnetic relaxation, ultimately leading to field-induced SMM behavior.

## Experimental Section

### General Information

Tetrahydrofuran (THF) was refluxed over potassium for several days and subsequently dried further over a Na/K alloy. Diethyl ether (Et_2_O) was dried by refluxing over a Na/K alloy. ^*n*^Hexane was dried over calcium hydride. In all cases, the solvents were tested for the presence of water and oxygen in the glovebox by the addition of one drop of potassium benzophenone radical solution to 2 mL of the solvent of interest. Terbium chloride (TbCl_3_) and (trimethylsilyl)­methyllithium (LiCH_2_SiMe_3_) solution in pentane (0.1 M) were purchased from Sigma-Aldrich and used as received. (Trimethylsilyl)­methylpotassium (KCH_2_SiMe_3_),^21^ tosyl azide (C_7_H_7_N_3_O_2_S),^22^ H_2_NAr*,^23^ KNHAr*,^24^ and potassium graphite (KC_8_)^25^ were synthesized according to literature procedures.

**Caution!** LiCH_2_SiMe_3_ solutions are pyrophoric and corrosive and must be handled by using proper needle and syringe techniques. C_7_H_7_N_3_O_2_S forms explosive mixtures with air at ambient temperatures. KC_8_ is a corrosive and extremely pyrophoric solid under ambient conditions. All manipulations were performed in an argon-filled MBRAUN glovebox with an atmosphere of <0.1 ppm of O_2_ and <0.1 ppm of H_2_O, and on the smallest practical scale following the procedures described below.

### Synthesis of (NHAr*)_2_TbCl, **1**

In a 4 mL vial, solid TbCl_3_ (168.8 mg, 0.636 mmol, 1 equiv) was cooled for 30 min at −78 °C in a cold well and was subsequently added to a 20 mL vial with a stirring, precooled (−78 °C) suspension of KNHAr* (681.5 mg, 1.272 mmol, 2 equiv) in 15 mL of Et_2_O. Subsequently, the reaction vessel was removed from the cold well and warmed to room temperature upon which the mixture gradually turned yellow, and a colorless precipitate formed, presumably potassium chloride. After stirring at room temperature for 16 h, the resulting cloudy yellow mixture was evaporated to dryness to yield yellow and colorless oily solids. These were triturated in ^*n*^hexane and dried under reduced pressure to yield powdery yellow and colorless solids. These solids were extracted with 18 mL of ^*n*^hexane to give a yellow mixture, which was filtered through Celite to remove colorless byproducts. The bright yellow filtrate was dried under dynamic vacuum to obtain amorphous yellow solids (quantitative crude yield). Yellow, block-shaped crystals of **1**, suitable for single-crystal X-ray diffraction analysis, were grown from a concentrated ^*n*^hexane solution at −35 °C after 3 d. The crystals were separated from the mother liquor and washed three times with cold ^*n*^hexane (∼1.5 mL each) and dried under reduced pressure for 1 h. Crystalline yield: 57% (430.3 mg, 0.362 mmol). Crystals of **1** are stable under an inert argon atmosphere at room temperature for several days. IR (FTIR, cm^–1^): 2958s, 2927m, 2867m, 1694w, 1602w, 1582w, 1460m, 1442w, 1410s, 1382m, 1361m, 1319m, 1255m, 1242m, 1166w, 1154w, 1100w, 1073m, 1005w, 957w, 938w, 923w, 876m, 847m, 832m, 796w, 777w, 750s, 728w, 654m. Anal. Calcd for C_72_H_100_N_2_ClTb: C, 72.80; H, 8.48; N, 2.36. Found: C, 73.15; H, 8.73; N, 2.35.

### Synthesis of (NHAr*)_2_Tb, **2**

In a 20 mL vial, crystalline solids of **1** (430.3 mg, 0.3622 mmol, 1 equiv) were dissolved in 8 mL of THF at −78 °C and were allowed to stir at −78 °C in a cold well for 30 min before KC_8_ (97.9 mg, 0.724 mmol, 2 equiv) was added directly to the stirring solution. An immediate color change from yellow to dark red was observed, followed by the formation of a dark black solid, presumably graphite, and potassium chloride. The reaction was subsequently diluted with an additional 3 mL of cold THF to ensure adequate stirring before being removed from the cold well and warming slowly to room temperature. After 1 h, the reaction mixture was evaporated to dryness under reduced pressure, and the dark red/brown residue was extracted in 18 mL of ^*n*^hexane, filtered, and dried under dynamic vacuum (quantitative crude yield). Dark brown, block-shaped crystals suitable for single-crystal X-ray diffraction analysis of **2** were obtained from a concentrated ^*n*^hexane solution at −35 °C after 3 d. The crystals were separated from the mother liquor and dried under a dynamic vacuum for 2 h. Crystalline yield: 14% (59.3 mg, 0.0515 mmol). Crystals of **2** are stored under an inert argon atmosphere at −35 °C, but are stable at room temperature for at least 24 h. Crystalline material of **2** degrades within 1 h under ambient atmosphere, even when covered in Paratone oil. IR (FTIR, cm^–1^): 2956s, 2925m, 2865m, 1603w, 1581w, 1565w, 1509w, 1459m, 1407s, 1380m, 1359s, 1317m, 1262s, 1186w, 1166w, 1154w, 1100w, 1065s, 1001m, 938w, 924w, 874m, 845m, 830m, 792w, 777w, 747s, 730m, 654m. Anal. Calcd for C_72_H_100_N_2_Tb·(C_4_H_8_O): C, 74.54; H, 8.89; N, 2.29. Found: C, 74.43; H, 9.29; N, 2.08.

### Single-Crystal X-ray Diffraction

Yellow and dark brown crystals of **1** and **2**, respectively, with dimensions of 0.328 × 0.245 × 0.107 mm^3^ and 0.136 × 0.075 × 0.053 mm^3^, respectively, were mounted on a nylon loop using Paratone oil. Data for **1** and **2** were collected on a XtaLAB Synergy, Dualflex, and HyPix diffractometer equipped with an Oxford Cryosystems low-temperature device, operating at *T* = 100.02(12) and 100.01(10) K, for **1** and **2**, respectively. Data for **1** and **2** were measured using ω scans using Mo Kα and Cu Kα radiation (microfocus sealed X-ray tube, 50 kV, 1 mA), respectively. The total number of runs and images was based on the strategy calculation from the program CrysAlisPro (Rigaku, V1.171.41.90a, 2020),^26^ which was used to retrieve and refine the cell parameters, as well as for data reduction. A numerical absorption correction based on Gaussian integration over a multifaceted crystal model empirical absorption correction using spherical harmonics was implemented in the SCALE3 ABSPACK scaling algorithm.^27^ The structures were solved in the *P*1̅ and *C*2/*c* space groups for **1** and **2**, respectively, by using intrinsic phasing with the ShelXL structure solution program.^28^ The structure was refined by least-squares using version 2018/2 of XL^28^ incorporated in Olex2.^29^ All non-hydrogen atoms were refined anisotropically. Hydrogen atom positions were calculated geometrically and refined by using the riding model.

### UV–Vis Spectroscopy

The UV–vis spectra were collected with an Agilent Cary 60 spectrometer at ambient temperature from 220 to 1100 nm. Samples were prepared in an argon-filled glovebox and filtered into 1 cm quartz cuvettes. The spectra were baseline corrected from a sample of dry Et_2_O.

### Infrared Spectroscopy

The IR spectra were recorded with an Agilent Cary 630 FTIR spectrometer on crushed crystalline solids under an inert nitrogen atmosphere.

### Elemental Analysis

Elemental analysis was carried out with a PerkinElmer 2400 Series II CHNS/O analyzer. The crystalline compounds of all samples (∼1–3 mg) were weighed into tin sample holders and folded multiple times to ensure proper sealing from the surrounding atmosphere. The samples were then transferred to the instrument in an airtight container.

### Magnetic Measurements

Magnetic susceptibility data were collected on a Quantum Design MPMS3 superconducting quantum interference device (SQUID) magnetometer. The magnetic samples of **1** and **2** were prepared by saturating and covering dried, crushed crystalline solids (**1**: 10.3 mg; **2**: 12.7 mg) with molten eicosane (**1**: 28.5 mg) at 60 °C or octadecane (**2**: 39.7 mg) at 40 °C to prevent crystallite torquing and to provide good thermal contact between the sample and the bath. The samples were sealed in an airtight container and transferred to the magnetometer. The core diamagnetism was estimated using Pascal’s constants.^30^

### *Ab Initio* Calculations

*Ab initio* calculations on **2** were carried out employing the ORCA 6.0.0 program suite^31–33^ using a complete active space self-consistent field (CASSCF)^34,35^ /*n*-valence electron perturbation theory (NEVPT2)^36–38^ approach. All calculations were accelerated by using the chain-of-spheres approximation (RIJCOSX).^39^

Coordinates obtained from single-crystal XRD were used, and the positions of the H atoms were refined via unrestricted DFT methods. For this, the Tb ion was replaced with diamagnetic Y, while all other non-H atoms were kept fixed. The TPSSh^40,41^ functional was used with the x2c-SVPall-2c^42,43^ basis set for peripheral C and H atoms and with x2c-TZVPall-2c^42,43^ for the Y atom and C/N atoms of the first coordination sphere alongside the x2c/J auxiliary basis set.^42,43^

A series of CASSCF calculations considering various active spaces (CAS­(9,7)–CAS­(9,16)) were conducted to scrutinize the inclusion of different metal or ligand orbitals in the active space (Tables S3–S6). Using the optimized coordinates with Y substituted for Tb and identical atomic basis sets (x2c-TZVPall-2c for Tb),^42,43^ with auxiliary basis automatically constructed (autoaux),^44^ these calculations were performed through averaging over 21 *S* = 7/2 roots. A final CASSCF/NEVPT2 calculation using the best CAS­(9,15) was carried out with 21 *S* = 7/2 and 21 *S* = 5/2 roots. Here, the fourth order reduced density matrix (4-RDM) was treated via the efficient implementation.^33,43^ For this last step, the Tb basis set was expanded to x2c-QZVPall-2c.^42,43^

Scalar relativistic effects were considered throughout via the exact two-component (x2c) Hamiltonian^45^ with diagonal local approximation to the unitary transformation matrix (DLU) approximation (DFT/CASSCF).^46^ Spin–orbit coupling effects were accounted for in the NEVPT2 step via quasi-degenerate perturbation theory (SOMF­(1x))^47,48^ with deactivated DLU. Picture change effects were included to compute the full relativistic Hamiltonian derivative. The Gaussian finite nucleus model was employed for the final single-point calculation.^49^

All calculations used improved integration grids (defgrid 3). The frozen core approximation was switched off for all CASSCF/NEVPT2 calculations (NoFrozenCore). Orbital visualizations of the CAS­(9,15) active orbitals were obtained via the VMD software (Figure 8).^50^

## Results and Discussion

Isolation of the terbium bis­(amide) chloride complex, (NHAr*)_2_TbCl (where Ar* = 2,6-(Ar′)_2_C_6_H_3_ with Ar′ = 2,4,6-(^*i*^Pr)_3_C_6_H_2_, **1**), proceeded through the reaction of TbCl_3_ with 2 equiv of the potassium *m*-terphenylamide salt, KNHAr*, in diethyl ether (Et_2_O) (Figure 2A). Yellow, block-shaped crystals of **1** suitable for single-crystal X-ray diffraction analysis were grown from a concentrated ^*n*^hexane solution at −35 °C over the course of 3 days in 57% crystalline yield (Figures 2B, S2, and S3). The six-coordinate Tb^III^ center adopts a distorted trigonal pyramidal geometry and is asymmetrically ligated by two NHAr*^–^ ligands, leading to a rare example of a Tb^III^ complex with an η^6^-coordinated arene moiety. In fact, the Tb–C distances of 2.817(2)–3.007(2) Å and Tb–Cnt (Cnt = centroid of the arene unit) distance of 2.556 Å are both consistent with the Tb–C and Tb–Cnt distances monitored for (C_6_Me_6_)­Tb­(AlCl_4_)_3_,^51^ and the bis­(*m*-terphenoxide)terbium tuck-in complex, (ArO)­Tb­(OAr″) (where Ar = 2,6-(2,6-(^*i*^Pr)_2_C_6_H_3_)­C_6_H_3_; Ar″ = 6-Dipp-2-(2-(^i^Pr)-6-CHMe­(CH_2_)­C_6_H_3_)­C_6_H_3_; Dipp = 2,6-(^i^Pr)_2_C_6_H_3_).^52^ By contrast, the pendant *m*-terphenyl moiety exhibits Tb–C_arene_ distances between 3.339(2) and 4.216(2) Å. The asymmetric binding of the NHAr* ligands in **1** to the terbium ion engenders inequivalent Tb–N distances (Å). The arene-coordinated NHAr* ligand exhibits a longer Tb–N distance of 2.257(1) Å in comparison to the Tb–N distance of 2.274(2) Å in the pendant *m*-terphenyl moiety. Here, the subtle change in metal–ligand distances can be attributed to the presence of a capping Tb–C_arene_ interaction, which may result in a less flexible Tb–N interaction. Additionally, the N–Tb–N angle of 136.7(1)° deviates from linearity owing to the presence of a ligating chloride anion, which bisects the Tb–N bond vectors, yielding Cl–Tb–N angles of 107°.

**Figure 2:**
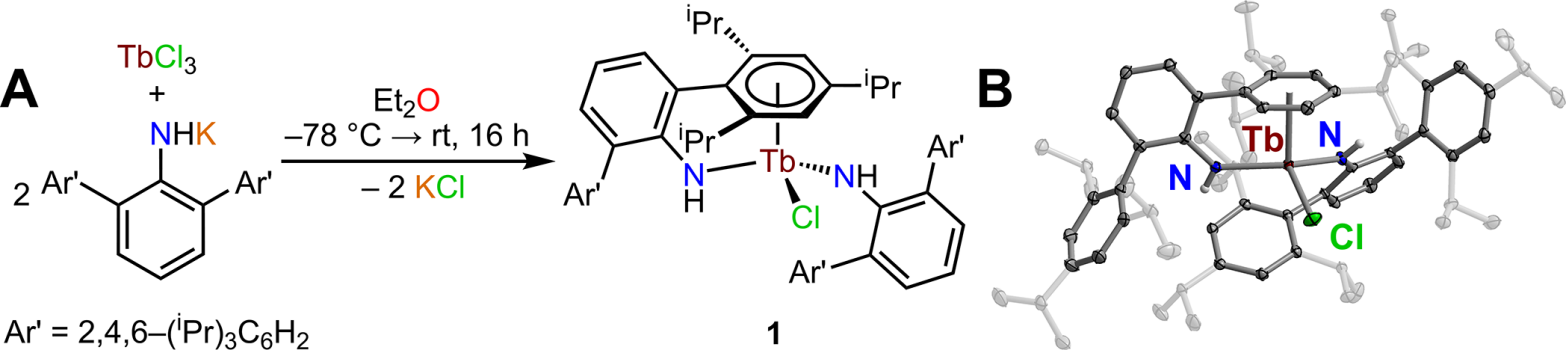
(A) Synthetic route for (NHAr*)_2_TbCl, **1**, where Ar* = 2,6-(Ar′)_2_C_6_H_3_ with Ar′ = 2,4,6-(^*i*^Pr)_3_C_6_H_2_. (B) Structure of (NHAr*)_2_TbCl, **1**, with thermal ellipsoids drawn at the 50% probability level. Dark red, green, blue, and gray ellipsoids represent Tb, Cl, N, and C atoms, respectively. White-gray spheres represent H atoms. H atoms bound to all carbon atoms have been omitted for clarity. The isopropyl substituents of the NHAr* ligands have been faded for clarity. Selected interatomic distances (Å) and angles (deg) for **1**: Tb–N = 2.274(2), 2.257(1); Tb–Cl = 2.547(1); Tb–Cnt = 2.556; N–Tb–N = 136.7(1).

Intriguingly, the chemical reduction of **1** with an excess of reducing agent, potassium graphite, in THF (Figure 3) forms a neutral terbium bis­(amide) complex, (NHAr*)_2_Tb, **2**, under extrusion of potassium chloride. Brown, block-shaped crystals of **2** suitable for single-crystal X-ray diffraction analysis were grown from a concentrated ^*n*^hexane solution at −35 °C over the course of 3 days in 14% crystalline yield (Figures S4 and S5). The attained molecular structure of **2** (Figure 3B) confirmed the generation of a rare example of a neutral, formally divalent Tb complex under displacement of the coordinating chloride anion, followed by precipitation of the alkali metal salt. **2** represents a terbium complex bearing formally the oxidation state of +2, which is rare for that ion. In fact, most molecular examples containing the highly reactive Tb^II^ ion have been tamed as salts of tris­(cyclopentadienyl),^3,10,19^ tris­(amide),^16^ and bis­(amidinate)^14^ scaffolds, placing **2** among the much smaller set of neutral divalent terbium complexes (Figure 1). The displacement of the chloride anion leads to a pronounced structural rearrangement of the NHAr* scaffolds, where the Tb^II^ center is sandwiched between two η^6^-coordinating arene moieties and two equatorially bound nitrogen atoms. The asymmetric unit of **2** features a terbium ion ligated by one NHAr* fragment and a noncoordinating THF molecule in the crystal lattice. Resultingly, the two Tb–N distances, as well as the Tb–C_arene_ interactions, are identical, owing to the crystallographic inversion center in **2**. Overall, **2** forms a distorted pseudotetrahedral geometry and constitutes the first formally divalent terbium complex featuring a metal–arene interaction. Intriguingly, **2** simultaneously represents the first crystallographic evidence for a bis­(η^6^-arene) sandwich complex of Tb in any oxidation state. The only terbium arene interactions that were crystallographically observed so far are within the terbocene complexes, (C_5_Me_4_R)_2_Tb­(η^2^-Ph)_2_BPh_2_ (where R = Me, H), which bear multiple Tb–arene interactions, albeit involving solely four carbon atoms of two phenyl rings belonging to a weakly coordinating tetraphenylborate anion.^11,53^ The Tb–C distances of **2** decrease marginally to 2.744(3)–3.026(2) Å with respect to **1**, giving rise to shortened Tb–Cnt distances of 2.484 Å. By contrast, the Tb–N distances in **2** increase slightly to 2.280(2) Å. More specifically, the Tb–N distance of 2.274(2) Å in the capping NHAr* ligand of **1** lengthens by 0.006 Å upon reduction and metathesis of the equatorial chloride ligand. Conversely, the N–Tb–N angle decreases dramatically to 102.4°. The change in crystallographic distances in **2** may be attributed to (a) the decreased Lewis acidity of Tb^II^, with respect to the highly-charged trivalent ion, leading to softer metal–ligand interactions, and (b) the extrusion of a coordinating chloride ion and reduction in steric bulk, which enables the formation of a bis­(arene) framework. A similar trend was observed in the neutral, divalent bis­(amidinate) terbium complex, (Piso)_2_Tb, following the reduction of the bis­(amidinate) terbium iodide precursor, (Piso)_2_TbI (where Piso = {(NDipp)_2_C^*t*^Bu}, Dipp = 2,6-(^*i*^Pr)_2_C_6_H_3_).^14^ as well as in the analogous yttrium congeners of **1** and **2**.^24^ A slight elongation in metal–ligand distances by 0.05 Å upon chemical reduction was monitored for the homoleptic [K­(crypt-222)]­[Tb­(N­(SiMe_3_)_2_)_3_] complex,^16^ albeit **1** undergoes pronounced changes in the primary coordination sphere following reduction. In sum, the elimination of the ligated chloride ligand through chemical reduction gives rise to a less sterically congested coordination sphere, which may enable the bulky NHAr* ligands to contact the Lewis acidic Tb center more closely.

**Figure 3:**
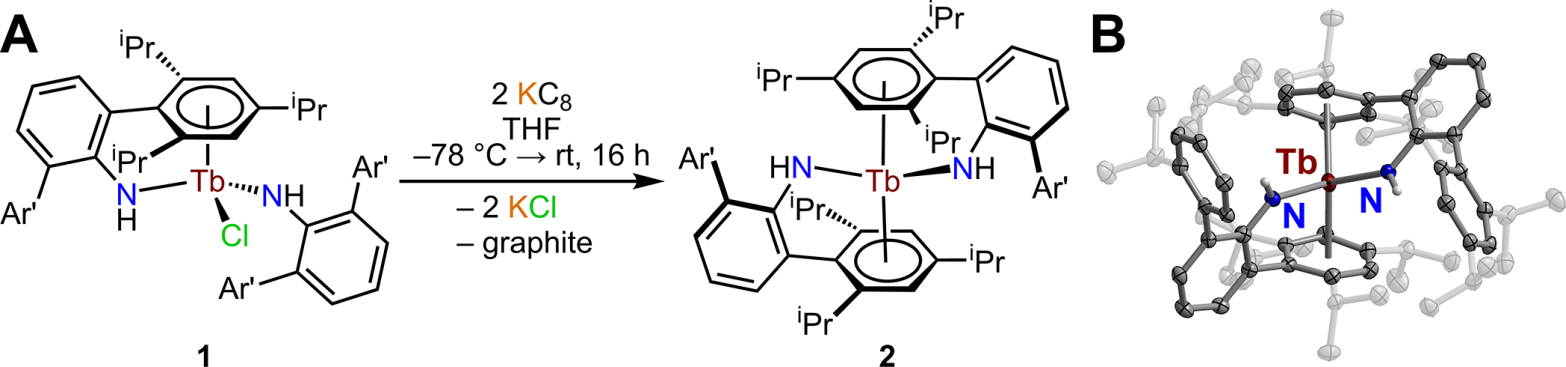
(A) Synthetic route for (NHAr*)_2_Tb, **2**. (B) Structure of **2** in a crystal of (NHAr*)_2_Tb·(OC_4_H_8_), with thermal ellipsoids drawn at the 50% probability level, where Ar* = 2,6-(Ar′)_2_C_6_H_3_ with Ar′ = 2,4,6-(^*i*^Pr)_3_C_6_H_2_. Dark red, blue, and gray ellipsoids represent Tb, N, and C atoms, respectively. White-gray spheres represent H atoms. H atoms bound to all carbon atoms and solvent molecules in the crystal lattice have been omitted for clarity. The isopropyl substituents of the NHAr* ligands have been faded for clarity. Selected interatomic distances (Å) and angles (deg) for **2**: Tb–N = 2.280(2); Tb–Cnt = 2.484; N–Tb–N = 102.3(1); Cnt–Tb–Cnt = 133.5.

**1** and **2** were also probed in the solid-state via infrared (IR) spectroscopy. The collected data reveal nearly superimposable vibrational spectra (Figures S9–S11). The high energy fingerprint regions exhibit ligand-centered vibrations, with pronounced N–H bending modes at 1582 and 1580 cm^–1^ for **1** and **2**, respectively.

To further elucidate the electronic structures, **1** and **2** were subjected to UV–vis spectroscopy (Figures 4, S6–S8). In ethereal solutions, strong absorption bands in the UV region were observed for **1** and **2** at 297 and 283 nm, respectively, which are ascribed to ligand-based π–π* transitions. Notably, **2** exhibits an additional lower energy transition at 315 nm and two broad absorption bands in the visible regime above 350 nm, which is ascribed to metal-based d-orbital transitions to d- and π*-orbitals.^3^ The emergence of d-orbital character in the absorption spectrum of **2** indicates a population of the 5d-manifold upon chemical reduction. However, the associated transitions may be weak, owing to the delocalization of the d-electron with ligand-based orbitals. A similar low energy feature was monitored for the analogous Y^II^ complex, (NHAr*)_2_Y, which further confirms the d-orbital character of **2**.^24^

**Figure 4:**
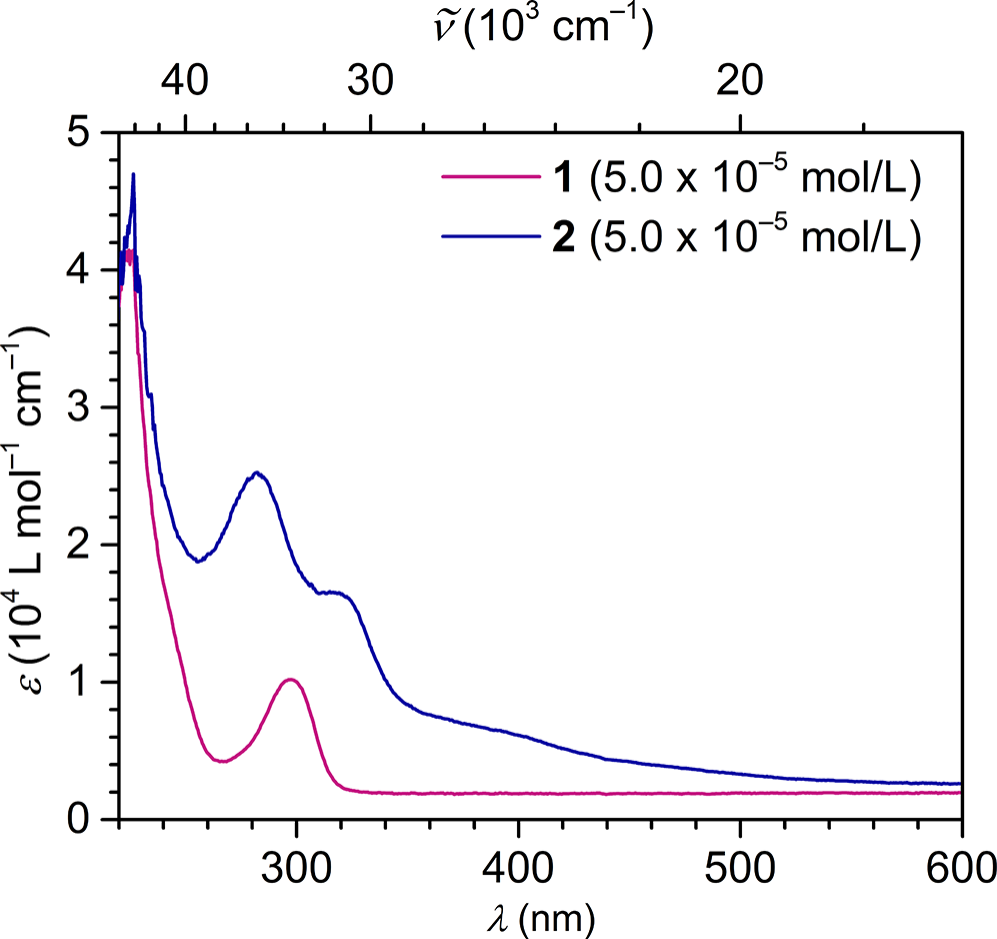
UV–vis spectra of (NHAr*)_2_TbCl, **1** (pink line), and (NHAr*)_2_Tb, **2** (dark blue line), recorded at 50 μmol/L concentrations in diethyl ether at room temperature.

The static magnetic properties of **1** were probed via direct-current (dc) magnetic susceptibility measurements from 2 to 300 K under a 0.1 T applied dc field. The room temperature molar magnetic susceptibility and temperature product (χ_M_ *T*) value of 11.83 cm^3^ K mol^–1^ is in excellent agreement with the expected value of an uncoupled trivalent terbium ion (Tb^III^ = ^7^F_6_, *S* = 3, *L* = 3, *J* = 6, *g* = 3/2, (χ_M_ *T*)_calc_ = 11.82 cm^3^ K mol^–1^)^54^ (Figures 5 and S12). As the temperature is lowered, the χ_M_ *T* value gradually declines to 9.53 cm^3^ K mol^–1^ at 10 K, which is followed by a downturn to 8.58 cm^3^ K mol^–1^ at 2 K. The decrease in the χ_M_ *T* value upon lowering the temperature is attributed to the depopulation of crystal field states. The field-dependent magnetization data (*M* vs *H*) were recorded between 2 and 10 K. At 2 K, the magnetization grows with rising field strength until it reaches a value of 5.10 μ_B_, whereas at higher temperatures, the magnetization increases rapidly without saturating (Figures S13 and S15). The reduced magnetization curves of **1** are nonsuperimposable, which alludes to the presence of pronounced magnetic anisotropy (Figure S14).

**Figure 5:**
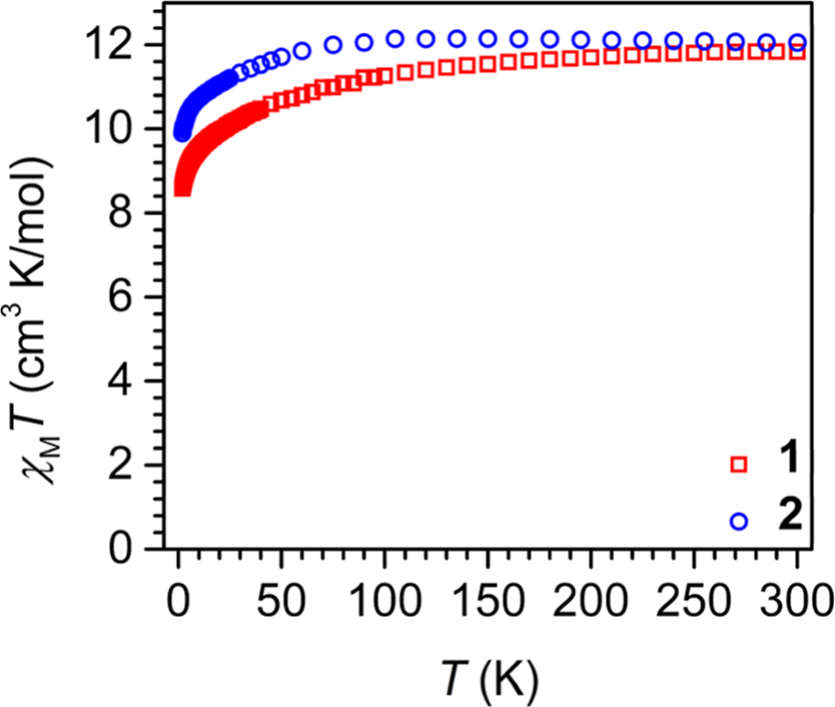
Variable-temperature dc magnetic susceptibility data for restrained polycrystalline samples of (NHAr*)_2_TbCl, **1** (red squares), and (NHAr*)_2_Tb, **2** (blue circles), collected under 0.1 T applied dc fields.

The magnetic properties of divalent Tb complexes are generally intricate to grasp due to system-dependent ambiguity regarding the orbitals housing the additional electron. While classical divalent ions such as Sm^II^, Eu^II^, and Yb^II^ are typically innate to a 4f^*n*+1^ configuration, the nonclassical divalent ions such as Tb^II^ have been shown to be better described through another configuration. For example, both the neutral amidinate Tb^II^ complex, (Piso)_2_Tb (Piso = {(NDipp)_2_C^*t*^Bu}, Dipp = 2,6-(^*i*^Pr)_2_C_6_H_3_), and the neutral cyclopentadienyl Tb^II^ complex, (Cp^*i*Pr5^)_2_Tb, exhibit a 4f^8^d^1^ configuration with a singly occupied d_*z* ^2^_-like {6s, 5d} hybrid orbital.^10,14,55^ The electronic situation may be even further complicated through magnetic coupling among the shells, which has a substantial impact on the magnetic properties: (A) the 4f–5d_*z* ^2^_ interaction is stronger than the spin–orbit coupling in the 4f shell. Here, the total spin *S*_tot_ = *S*_4f_ + 1/2 couples to the 4f orbital angular momentum *L*_4f_, resulting in the value *J*_tot_. (B) 4f–5d_*z* ^2^_ interaction is weaker, affording *J*_4f_ + *s*(1/2). The collection of magnetic susceptibility data to canvas the temperature dependence of the product of molar magnetic susceptibility and temperature (*χ*_M_ *T*) can serve as an experimental means to distinguish between the coupling schemes. In the case of (A), a value of 14.42 cm^3^ K mol^–1^ is expected, while case (B) demands a value of 12.19 cm^3^ K mol^–1^.^1^ Of note, for both the mentioned complexes, the 4f–5d_*z* ^2^_ coupling was found to be dominant.

Static magnetic susceptibility measurements on a polycrystalline sample of **2** were carried out between 2 and 300 K under a 0.1 T dc field (Figure S16). At 300 K, the *χ*_M_ *T* value of 12.05 cm^3^ K mol^–1^ is slightly larger than the expected value of 11.81 cm^3^ K mol^–1^ for a single Tb^III^ ion (4f^8^) and is substantially smaller than the anticipated value of 14.17 cm^3^ K mol^–1^ for a Tb^II^ ion (4f^9^), effectively ruling out the population of the 4f shell. In contrast to the other neutral Tb^II^ complexes, the room temperature χ_M_ *T* value is considerably smaller, where 12.6 cm^3^ K mol^–1^ was found for (Piso)_2_Tb and 12.72 cm^3^ K mol^–1^ was observed for (Cp^*i*Pr5^)_2_Tb. These values vary from the expected value of 14.42 cm^3^ K mol^–1^ for a strong 4f–5d_*z* ^2^_ interaction, where such deviation of the χ_M_ *T* value for divalent lanthanide complexes has been rationalized to originate from the magnitude in 4f–5d_*z* ^2^_ coupling (Ω).^14^ Here, Ω describes the isotropic f–d coupling parameter with negative values indicating the parallel alignment of the d and f spins. For **2**, the room temperature χ_M_ *T* value matches with vanishingly low Ω values of 12.19 cm^3^ K mol^–1^, which would correspond to a differing electronic structure relative to the amidinate and cyclopentadienide complexes shown in Figure 1. Accordingly, the additional electron is likely not residing in a single 5d/6s hybrid orbital but is rather found in multiple degenerate hybrid orbitals, potentially even with significant ligand contributions.

Tb^II^ is a Kramers ion, innate to a doubly degenerate ground state, which is a prerequisite for a complex to exhibit single-molecule magnet (SMM) behavior. SMMs are intriguing for their potential applications in conductive materials,^56,57^ magnetic refrigeration,^58,59^ and quantum information technologies.^60–65^ The terbium ion among all lanthanide ions was found to produce the first SMMs comprising only one metal center, concomitant with the discovery of the first lanthanide-based SMMs.^66^ The breakthrough advance achieved with the Tb^III^ phthalocyanine SMM was attributed to the large magnetic anisotropy associated with the Tb^III^ ion which, despite its non-Kramers nature, showed slow magnetic relaxation due to a doubly degenerate ground state induced by a strongly axial ligand field.^66^ In principle, the Kramers nature of the Tb^II^ ion in **2** could result in slow magnetic relaxation. Inspired by this prospect, the dynamic magnetic properties of **2** were probed via variable-temperature, variable-frequency alternating current (ac) magnetic susceptibility measurements (Figure 6B). In the absence of an external dc magnetic field, no peaks in the out-of-phase ac susceptibility (χ_M_″) were observed. This may stem from prevalent ground-state quantum tunneling of magnetization (QTM). This relaxation process can be suppressed via application of an external dc magnetic field during ac measurements.^54,67^ The field dependence of the χ_M_″ signal was explored by applying dc fields between 500 and 2000 Oe at 1.8 K, where the optimum field was determined to be 1250 Oe (Figures S17 and S18). Upon application of this optimum 1250 Oe dc field, peaks emerged between 1.8 and 16 K. The relaxation times, τ, at each temperature were obtained by fitting the vs χ_M_″ (Cole–Cole) plots via a Cole–Davidson model as implemented in CCFit2 (Figures 6 and S19).^68^ The extracted τ values were used to construct the Arrhenius plot (ln­(τ) vs 1/*T*) and were subsequently fitted to a Raman and an Orbach relaxation process according to $\tau^{-1}=CT^{n}+\tau_{0}^{-1}\;\exp\left(-\frac{U_{eff}}{k_{B}T}\right)$ , where *C* and *n* are parameters of the Raman relaxation mechanism. The best fit yielded an effective barrier to spin relaxation of *U*_eff_ = 105(3) cm^–1^ and an attempt time of τ_0_ = 3.2(7) × 10^–9^ s with *C* of 1.2(1) × 10^1^ s^–1^ K^*–n*^ and *n* of 1.27(5) (Figures S20 and S21, Table S2). The obtained *n* value is lower compared to those of other SMMs.^14,69–71^ Low *n* values may arise from an operative Direct relaxation process, as has been observed for select SMMs where ac data collection proceeded under an applied dc magnetic field.^67,72^ To probe this possibility for **2**, the extracted relaxation times were fit to a Direct and Orbach process according to $\tau^{-1}=AH^{x}T+\tau_{0}^{-1}\;\exp\left(-\frac{U_{eff}}{k_{B}T}\right)$ , where *A* and *x* are the freely refined Direct relaxation parameters, with *x* adopting values of 2 for non-Kramers ions and 4 for Kramers ions.^67^ However, fitting the temperature-dependent τ for **2** does not require the field as the fieldis static and temperature-independent, simplifying the Direct relaxation expression to *AT*. Notably, employing these two relaxation processes yielded no satisfactory fit of the experimental data (Figure S22 and Table S2). The consideration of all three relaxation mechanisms, namely a Direct, a Raman, and an Orbach relaxation process according to $\tau^{-1}=AT+CT^{n}+\tau_{0}^{-1}\;\exp\left(-\frac{U_{eff}}{k_{B}T}\right)$ afforded for the best fit an *U*_eff_ of 112(5) cm^–1^, a τ_0_ of 1.7(7) × 10^–9^ s, a *C* of 5(5) × 10^–2^ s^–1^ K^–*n*^, and a slight improvement in *n* to 4(1) (Figures 6 and S23 and Table S2). Due to the scarcity of divalent Tb complexes and the even rarer occurrence of magnetic relaxation analyses on them, these values can only be meaningfully compared to the two other neutral, divalent Tb complexes (Cp^*i*Pr5^)_2_Tb and (Piso)_2_Tb.^10,14^ The obtained *U*_eff_ of 112(5) cm^–1^ for **2** is lower than the corresponding values obtained for these complexes and likely arises from the distinctly different electronic structure of **2**. The lack of magnetic relaxation data for ionic divalent complexes precludes any further comparisons.^3,16,17,73^

**Figure 6:**
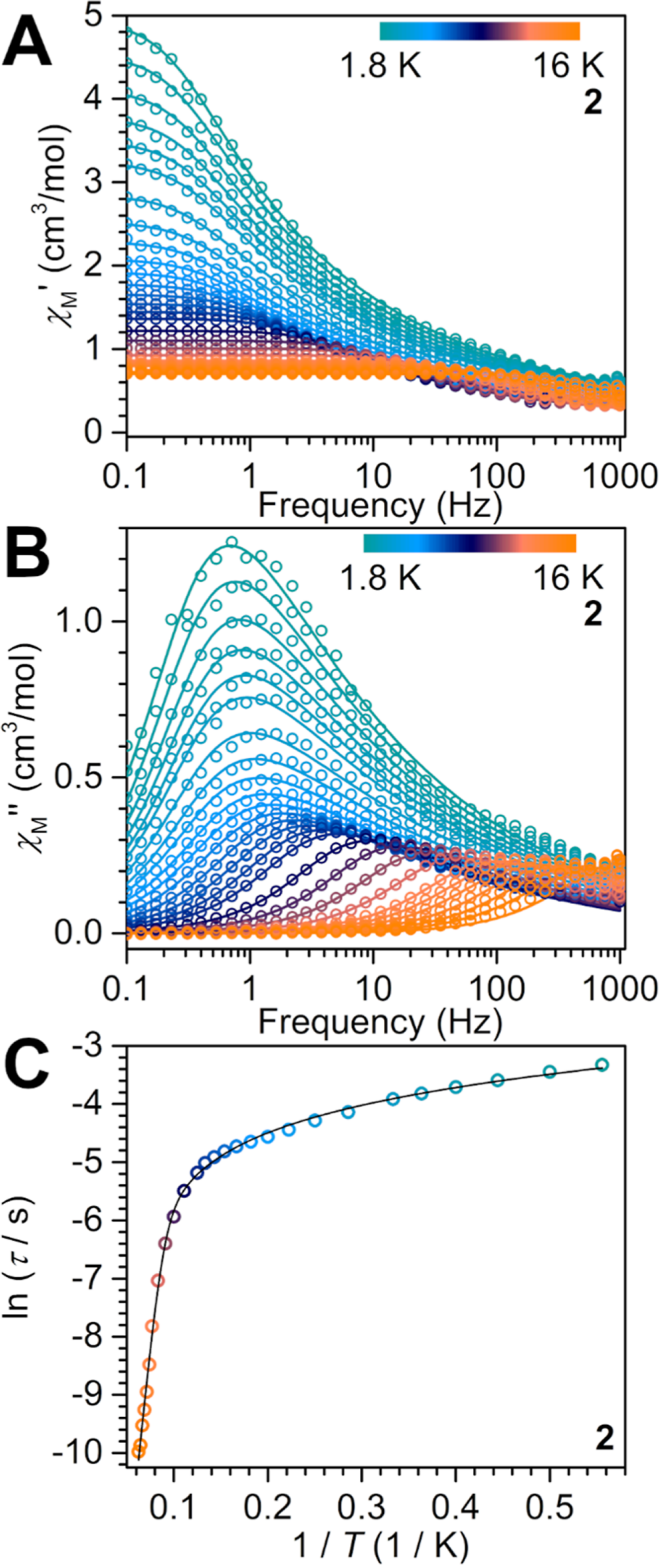
Variable-temperature, variable-frequency in-phase (A) and out-of-phase (B) ac magnetic susceptibility data collected under a 1250 Oe applied dc field for (NHAr*)_2_Tb, **2**, from 1.8 to 16 K. Solid lines indicate the fits to the Cole–Davidson model. Plot of the natural log of the relaxation time, τ (turquoise to orange circles), vs the inverse temperature for **2** (Arrhenius plot (C)). The black line corresponds to a fit of the data in the temperature range of 1.8–16 K to an Orbach, a Raman, and a Direct process yielding *U*_eff_ = 112(5) cm^–1^, τ_0_ = 1.7(7) × 10^–9^ s, *C* = 5(5) × 10^–2^ s^–1^ K^–*n*^ and *n* = 4(1), and *A* = 1.6(7) × 10^1^ s^–1^ K^–1^. The deconvolution is shown in Figure S23.

To further explore the magnetization characteristics, field-dependent magnetization (*M* vs *H*) experiments were performed at 1.8 K employing a 100 Oe/s scan rate. Scans from +7 to −7 T revealed superimposable curves without any remanent magnetization, suggestive of strong quantum tunneling of the magnetization. This interpretation is in accordance with the predominant QTM observed on the timescale of the conducted ac magnetic measurements under zero dc field (Figure S25). At 7 T, a maximum magnetization value of 4.526 μ_B_ is reached without achieving saturation. Interestingly, this value is very close to the ∼4.2 μ_B_ *M*_max_ value found for (Piso)_2_Tb despite their seemingly different electronic structures.^14^ The reduced magnetization curves (*M* vs *H*/*T*) are nonsuperimposable, indicative of strong magnetic anisotropy (Figure 7).

**Figure 7:**
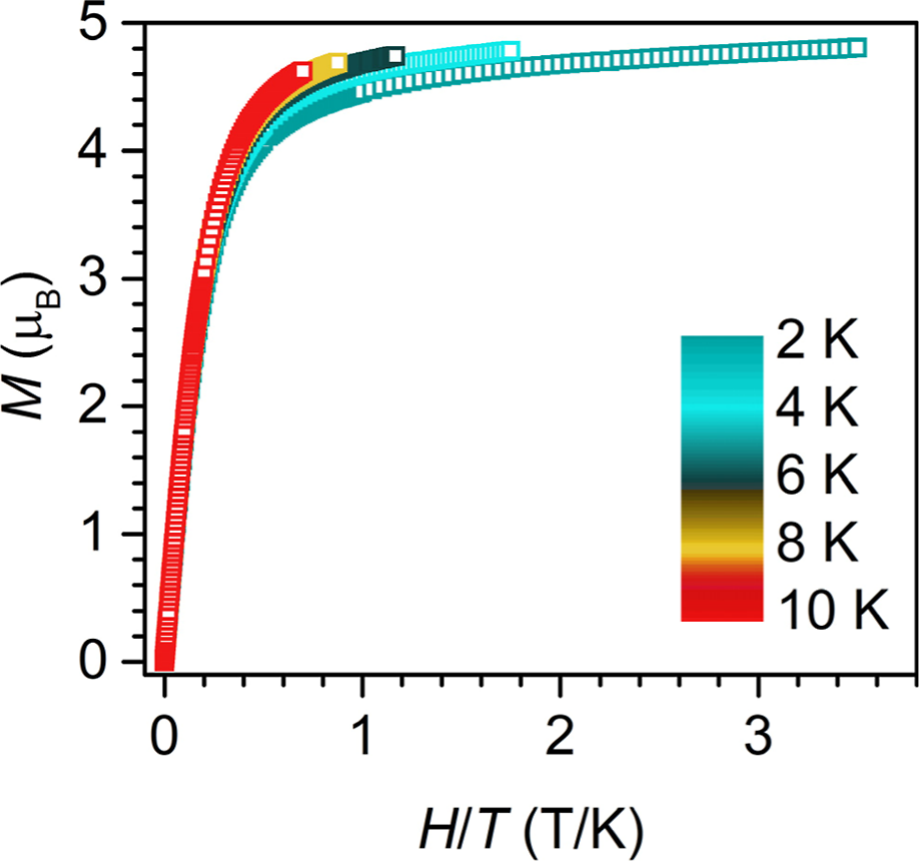
Reduced magnetization data of (NHAr*)_2_Tb, **2**, collected from 0 to 7 T at 2, 4, 6, 8, and 10 K.

To elucidate the electronic structure of **1**, we carried out *ab initio* calculations using the Orca 6.0.0 program suite. A complete active space self-consistent field (CASSCF)/*n*-valence electron perturbation theory (NEVPT2) approach was chosen considering the Tb 4f-, 5d-, and the three lowest-lying ligand-based π* orbitals in a (9,15) active space (Tables S3–S6). Intriguingly, across all other tested active space combinations, the additional electron was exclusively found to reside in the primarily ligand-based π* orbitals, which gain up to a maximum 15% Tb 5d contribution upon orbital optimization (Figure 8, Table S3). The atomic character of the Tb 5d orbitals is largely retained while remaining essentially unoccupied. A similar bonding situation was previously found via DFT calculations on the Y congener of **2**, (NHAr*)_2_Y.^24,74^ Inclusion of additional virtual metal- or ligand-based orbitals such as Tb 6s- or higher-lying π*-orbitals did not meaningfully change the low-lying energy spectrum and was therefore not further accounted for (Table S5). The inclusion of dynamic correlation correction via NEVPT2 and spin–orbit coupling via quasi-degenerate perturbation theory (QDPT) uncovered the first excited state at 160.8 cm^–1^ (Table S7). This energy is within the same order of magnitude as the experimentally determined *U*_eff_ but ∼44% larger, which may be ascribed to the small magnitude of experimentally determined *U*_eff_ itself and the associated larger impact of calculation accuracy. Alternatively, the deviation may be due to the fact that the calculated electronic structure may suffer from insufficiently accounting for dynamic electron correlation.^75^ This is somewhat considered by the NEVPT2 correction to the CASSCF-obtained states, but it cannot recover the entire correlation energy and leaves room for optimization.

**Figure 8:**
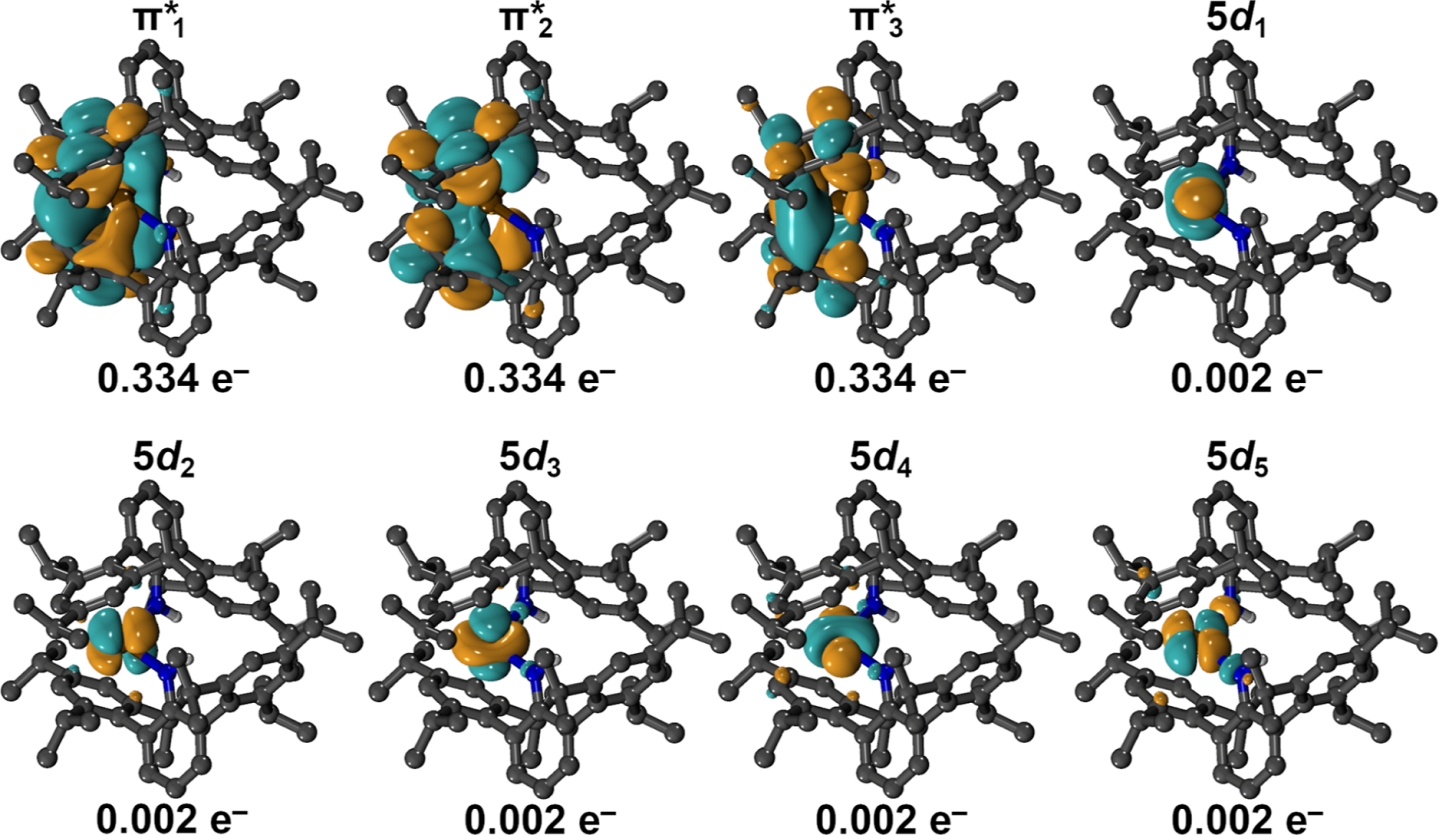
Orbital depictions of the active space CASSCF natural orbitals and respective occupations of the final CAS­(9,15) (isovalue: 0.03) for (NHAr*)_2_Tb, **2** (summarized in Table S3). The seven 4f orbitals are occupied by eight electrons, in accordance with a Tb^III^ ion, and therefore omitted for clarity (average orbital occupation: 1.143).

The inclusion of additional occupied ligand partner orbitals to the occupied π orbitals or a second shell of virtual f/d orbitals might deliver sufficient variability to recover the electron–electron correlation energy to a fuller extent and, thus, reduce the energy of low-lying states. However, due to prohibitive computational cost and general CASSCF convergence difficulties in the presence of multiple uncorrelated (doubly- or unoccupied) orbitals in the active space, such approaches were abstained from.

In sum, the absence of out-of-phase (χ_M_″) ac magnetic susceptibility signals under zero applied dc field and the superimposable forward and backward scans in the field-dependent magnetization experiments allude to QTM being the dominant relaxation mechanism in **2**. The proximity of *U*_eff_ and the calculated first excited state in terms of magnitude and value further allows the conclusion that the magnetic relaxation in **2** occurs through the first excited state in the presence of an external magnetic field. Furthermore, our calculations confirm that the additional electron in **2** introduced through chemical reduction of **1** results in a situation that may be described as a ligand-based organic radical coordinated to a Tb^III^ ion, rather than a true Tb^II^ ion.

## Conclusion

In conclusion, we have synthesized, isolated, and characterized two unprecedented terbium bis­(amide) complexes. The first compound is a half-sandwich-like bis­(amide)­(η^6^-arene) Tb^III^ chloride complex, (NHAr*)_2_TbCl, **1**, and the second is a neutral {bis­[(amide)-(η^6^-arene)]} Tb^II^ sandwich complex, (NHAr*)_2_Tb, **2**, comprising the rare, formally divalent oxidation state for terbium. Remarkably, the spectroscopic and magnetic characterization of **2** revealed that the compound contains a formally divalent terbium ion since the additional electron is found to reside in three primarily arene ligand-based hybrid orbitals, as corroborated by *ab initio* calculations. Thus, **2** constitutes a scarce and isolable example of a Tb^II^ complex. Single-crystal X-ray diffraction and UV–vis spectroscopic analysis indicate an oxidation state change following chemical reduction of **1**. Magnetic measurements of **2** revealed distinct properties, in fact, differing from those of the few other Tb^II^ complexes known. **2**, containing a Kramers ion, exhibits slow magnetic relaxation under an applied dc field and, thus, can be placed among a very small set of Tb^II^-based single-molecule magnets. The magnetic data of **2** further suggest the presence of a uniquely distinct set of hybrid orbitals containing the additional electron, which is clearly different from the reported 4f/6s hybrid orbitals in other divalent Tb complexes. Given the scarcity of isolable and stable Tb^II^ complexes, our study represents an important contribution for the prediction and understanding of the magnetic properties of divalent terbium complexes.

## Supplementary Material



## Notes

Spectroscopic and magnetic data, as well as detailed crystallographic information for **1** and **2** (PDF)
