# Supplementary material for: Slow Magnetic Relaxation in a Rare, Neutral, Formally Divalent Terbium Bis(amide) Complex
Source: Inorg Chem. 2025 May 20;64(21):10359–68. doi: 10.1021/acs.inorgchem.4c05349 (PMC12135042; doi:10.1021/acs.inorgchem.4c05349)
Supplement: Supplementary file 1 [file ic4c05349_si_001.pdf]

Supporting Information

for

Slow Magnetic Relaxation in a Rare, Neutral, Formally Divalent Terbium Bis(amide) Complex

Florian Benner, Elizabeth R. Pugliese, Ernesto Castellanos, Saroshan Deshapriya, and Selvan Demir*

Department of Chemistry, Michigan State University, 578 South Shaw Lane, East Lansing, Michigan 48824, USA

*Correspondence to: sdemir@chemistry.msu.edu (S.D.)

Table of Contents

Figure S1. Selected examples of divalent Tb complexes. **S4**

1 X-ray Crystallography **S5**

Table S1. Crystallographic data and structural refinements of $(NHAr^*)_2TbCl$, **1**, and $(NHAr^*)_2Tb$, **2**. **S5**

Figure S2. Structure of $(NHAr^*)_2TbCl$, **1**. **S6**

Figure S3. Space filling model of $(NHAr^*)_2TbCl$, **1**. **S7**

Figure S4. Structure of $(NHAr^*)_2Tb$, **2**. **S8**

Figure S5. Space filling model of $(NHAr^*)_2Tb$, **2**. **S9**

2 UV-vis Spectroscopy **S10**

Figure S6. UV-vis absorption spectra of $(NHAr^*)_2TbCl$, **1**. **S10**

Figure S7. UV-vis absorption spectra of $(NHAr^*)_2Tb$, **2**. **S11**

Figure S8. UV-vis absorption spectrum of $(NHAr^*)_2Tb$, **2**, recorded at 500 µmol/L. **S12**

3 IR Spectroscopy **S13**

Figure S9. FTIR spectra of $(NHAr^*)_2TbCl$, **1**, and $(NHAr^*)_2Tb$, **2**. **S13**

Figure S10. FTIR spectrum of $(NHAr^*)_2TbCl$, **1**. **S14**

Figure S11. FTIR spectrum of $(NHAr^*)_2Tb$, **2**. **S15**

4 Magnetic Measurements **S16**

Figure S12. Variable-temperature dc magnetic susceptibility data of $(NHAr^*)_2TbCl$, **1**. **S16**

Figure S13. Field dependent magnetization data for $(NHAr^*)_2TbCl$, **1**, collected from 0 to 7 T between 2 K and 10 K. **S17**

Figure S14. Field dependent reduced magnetization data for $(NHAr^*)_2TbCl$, **1**, collected from 0 to 7 T between 2 K and 10 K. **S18**

Figure S15. Plot of magnetization (*M*) vs dc magnetic field (*H*) for $(NHAr^*)_2TbCl$, **1**. **S19**

Figure S16. Variable-temperature dc magnetic susceptibility data of $(NHAr^*)_2Tb$, **2**. **S20**

Figure S17. In-phase (χ_M') and out-of-phase (χ_M'') components of the ac magnetic susceptibility for $(NHAr^*)_2Tb$, **2**, at 1.8 K under dc fields ranging from 0 Oe to 2000 Oe. **S21**

Figure S18. Plot of the natural log of the relaxation time, τ, versus the applied magnetic field obtained from ac measurements, for $(NHAr^*)_2Tb$, **2**, at 1.8 K. **S22**

Figure S19. Cole-Cole (Argand) plots for ac susceptibility collected under 1250 Oe applied dc field for $(NHAr^*)_2Tb$, **2**. **S23**

Figure S20. Individual contributions of the multiple magnetic relaxation pathways to the Arrhenius plot of $(NHAr^*)_2Tb$, **2**, at 1250 Oe with an Orbach and a Raman process. **S24**

Figure S21. Arrhenius plot of the natural log of the relaxation time, τ, versus the inverse temperature obtained from ac measurements, for $(NHAr^*)_2Tb$, **2**. **S25**
Figure S22. Individual contributions of the multiple magnetic relaxation pathways to the Arrhenius plot of $(NHAr^*)_2Tb$, **2**, at 1250 Oe with an Orbach and a Direct process. **S26**
Figure S23. Individual contributions of the multiple magnetic relaxation pathways to the Arrhenius plot of $(NHAr^*)_2Tb$, **2**, at 1250 Oe with an Orbach, a Raman, and a Direct process. **S27**
Table S2. Best-fit parameters for the relaxation times of **2** considering different relaxation processes. **S27**
Figure S24. Field dependent magnetization data for $(NHAr^*)_2Tb$, **2**, collected from 0 to 7 T between 2 K and 10 K. **S28**
Figure S25. Plot of magnetization (*M*) vs dc magnetic field (*H*) at an average sweep rate of 100 Oe/s for $(NHAr^*)_2Tb$, **2**, at 1.8 K. **S29**

5 *Ab Initio* Calculations **S30**

Table S3. State-average occupation numbers of the optimized orbitals in different CASSCF calculations of **2**. **S31**
Table S4. Single point energies of each converged active space calculation. **S31**
Table S5. CASSCF state-averaged energies for the 21 S = 7/2 states. **S32**
Table S6. Relative CASSCF state-averaged energies for the 21 S = 7/2 and 21 S = 5/2 states. **S33**
Table S7. Relative energies of the 50 lowest-lying CASSCF(9,15)/NEVPT2/QDPT states. **S34**

6 References **S35**

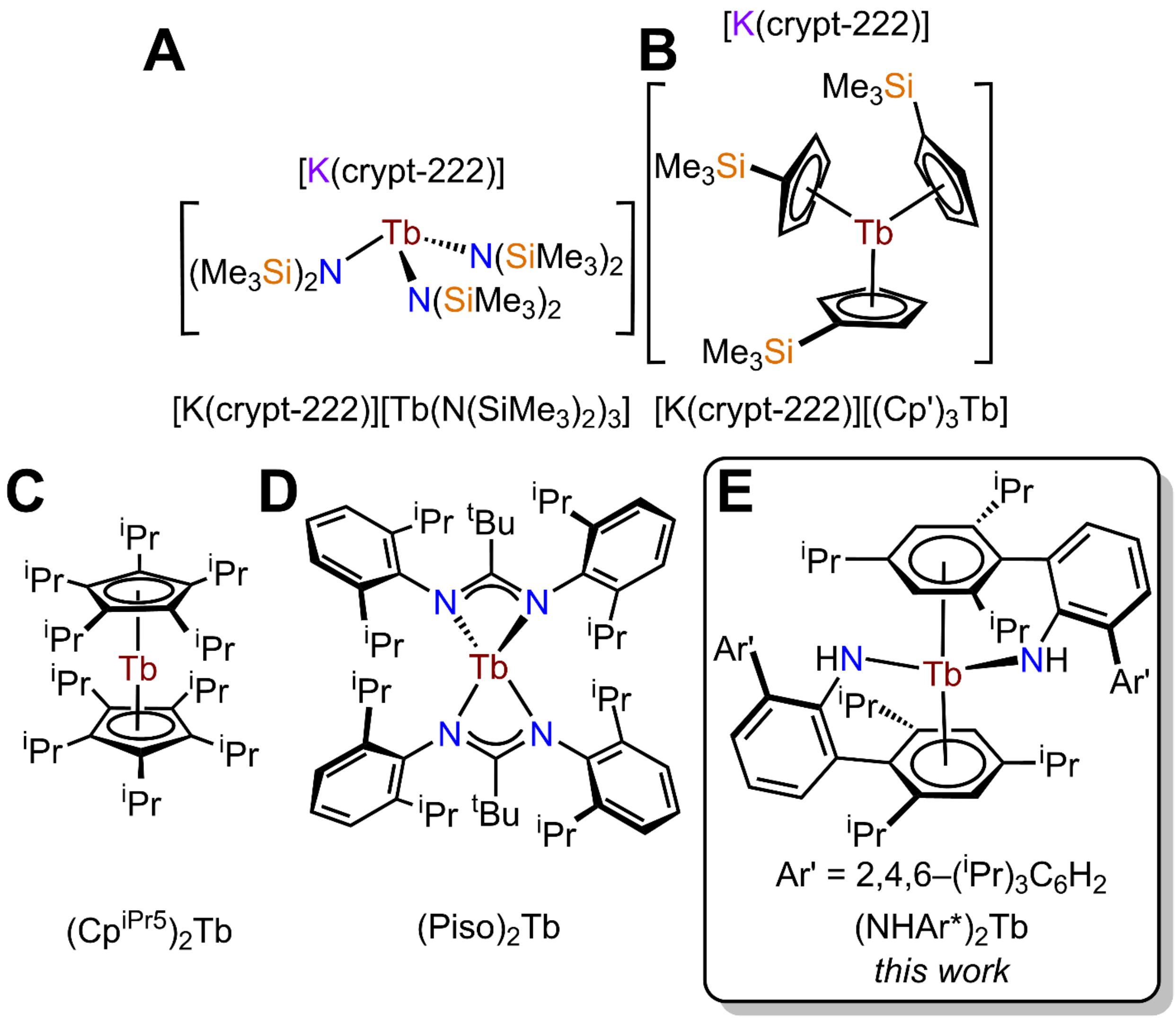

Figure S1. Selected examples of crystallographically characterized Tb^{II} complexes. (Top) Examples of anionic, divalent Tb complexes (**A**) [K(crypt-222)][Tb(N(SiMe$_3$)$_2$)$_3$],[1] and (**B**) [K(crypt-222)][(Cp')$_3$Tb] (where Cp' = $C_5H_4SiMe_3$).[2] (Bottom) Examples of neutral, divalent Tb complexes (**C**) (Cp^{iPr5})$_2$Tb,[3] (Piso)$_2$Tb (where Piso = {N(2,6-(^{i}Pr)$_2$C$_6$H$_3$)}$_2$C^{t}Bu),[4] and (NHAr*)$_2$Tb (where Ar* = 2,6-(Ar')$_2$C$_6$H$_3$, Ar' = 2,4,6-(^{i}Pr)$_3$C$_6$H$_2$).

1 X-ray Crystallography

Table S1. Crystallographic data and structural refinements of $(NHAr^*)_2TbCl$, **1**, and $(NHAr^*)_2Tb$, **2**, where Ar* = 2,6-$(Ar')_2C_6H_3$, Ar' = 2,4,6-$(^iPr)_3C_6H_2$. **2** crystallized with one tetrahydrofuran solvent molecule in the lattice as: $(NHAr^*)_2Tb\cdot(OC_4H_8)$.

	**1**	**2**
CCDC no.	2404189	2404190
Empirical formula	$C_{72}H_{100}ClN_2Tb$	$C_{76}H_{108}N_2OTb$
Formula weight	1187.90	1224.56
Temperature/K	100.02(12)	100.01(10)
Crystal system	triclinic	monoclinic
Space group	P-1	C2/c
a/Å	12.67120(10)	18.03210(10)
b/Å	14.6457(2)	17.00880(10)
c/Å	18.6691(2)	22.8087(2)
α/°	102.0630(10)	90
β/°	102.2880(10)	106.3010(10)
γ/°	98.6710(10)	90
Volume/Å^3	3240.62(6)	6714.31(9)
Z	2	4
ρ_{calc}g/cm^3	1.217	1.211
μ/mm^{-1}	1.173	5.496
F(000)	1256.0	2604.0
Crystal size/mm^3	0.328 × 0.245 × 0.107	0.136 × 0.075 × 0.053
Radiation	Mo Kα (λ = 0.71073)	CuKα (λ = 1.54184)
2*Θ* range for data collection/°	5.52 to 63.568	7.288 to 160.49
Index ranges	-18 ≤ h ≤ 17, -21 ≤ k ≤ 21, -27 ≤ l ≤ 26	-22 ≤ h ≤ 22, -21 ≤ k ≤ 20, -29 ≤ l ≤ 28
Reflections collected	60417	70012
Independent reflections	18284 [R_{int} = 0.0370, R_{sigma} = 0.0394]	7301 [R_{int} = 0.0369, R_{sigma} = 0.0164]
Data/restraints/parameters	18284/0/736	7301/66/373
Goodness-of-fit on F^2	1.057	1.037
Final R indexes [I>=2σ (I)]	R_1 = 0.0276, wR_2 = 0.0645	R_1 = 0.0372, wR_2 = 0.1028
Final R indexes [all data]	R_1 = 0.0323, wR_2 = 0.0663	R_1 = 0.0376, wR_2 = 0.1032
Largest diff. peak/hole / e Å^{-3}	0.98/-0.68	1.38/-0.69

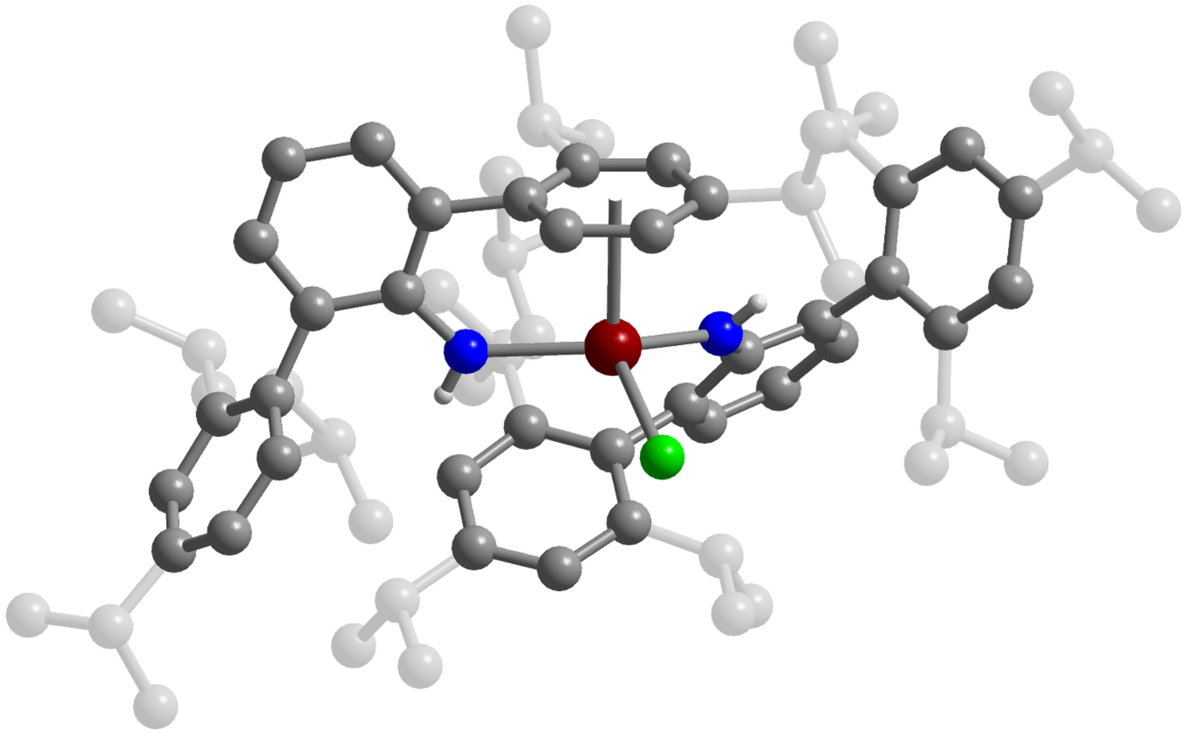

Figure S2. Structure of $(NHAr^*)_2TbCl$, **1**, where Ar* = 2,6-$(Ar')_2C_6H_3$, Ar' = 2,4,6-$(^iPr)_3C_6H_2$. Dark red, green, blue, gray, and white-gray spheres represent Tb, Cl, N, C, and H atoms, respectively. H atoms bound to all carbon atoms have been omitted for clarity. The isopropyl substituents of the NHAr* ligands have been faded for clarity.

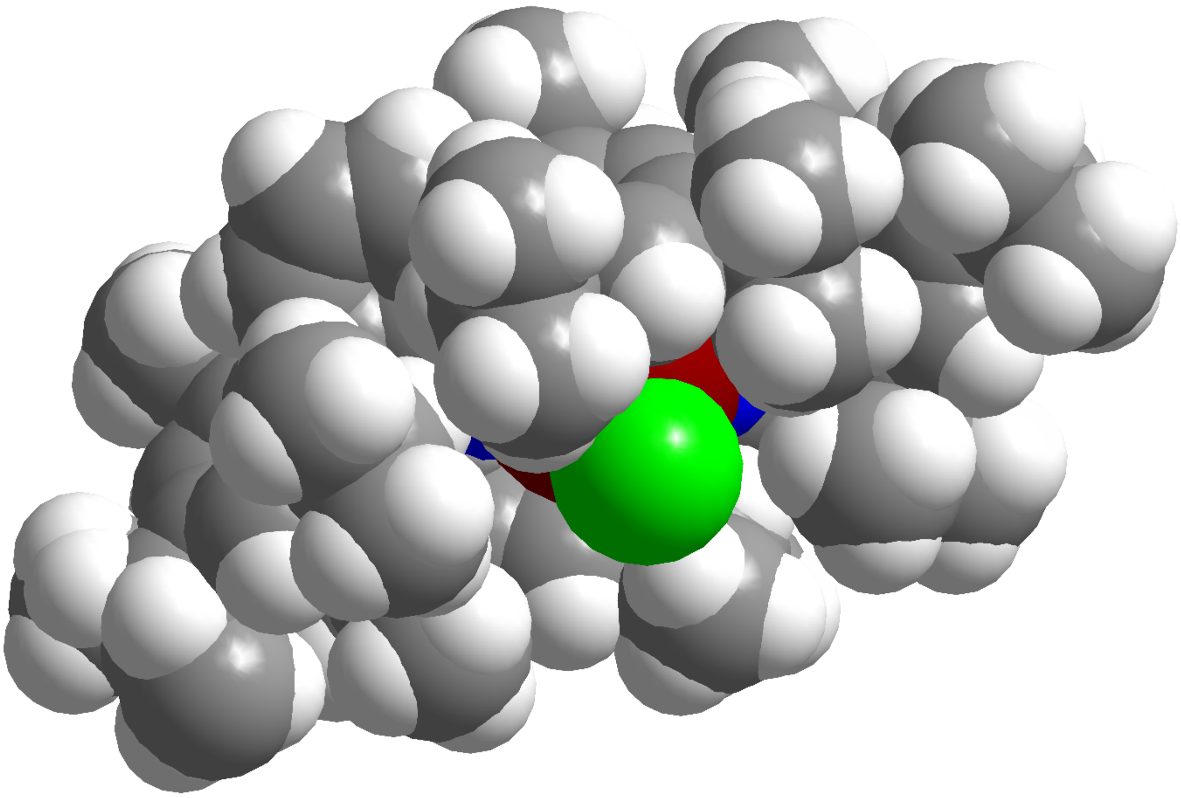

Figure S3. Space filling model of $(NHAr^*)_2TbCl$, **1**, where Ar* = 2,6-$(Ar')_2C_6H_3$, Ar' = 2,4,6-$(^iPr)_3C_6H_2$. Dark red, green, blue, gray, and white-gray spheres represent Tb, Cl, N, C, and H atoms, respectively.

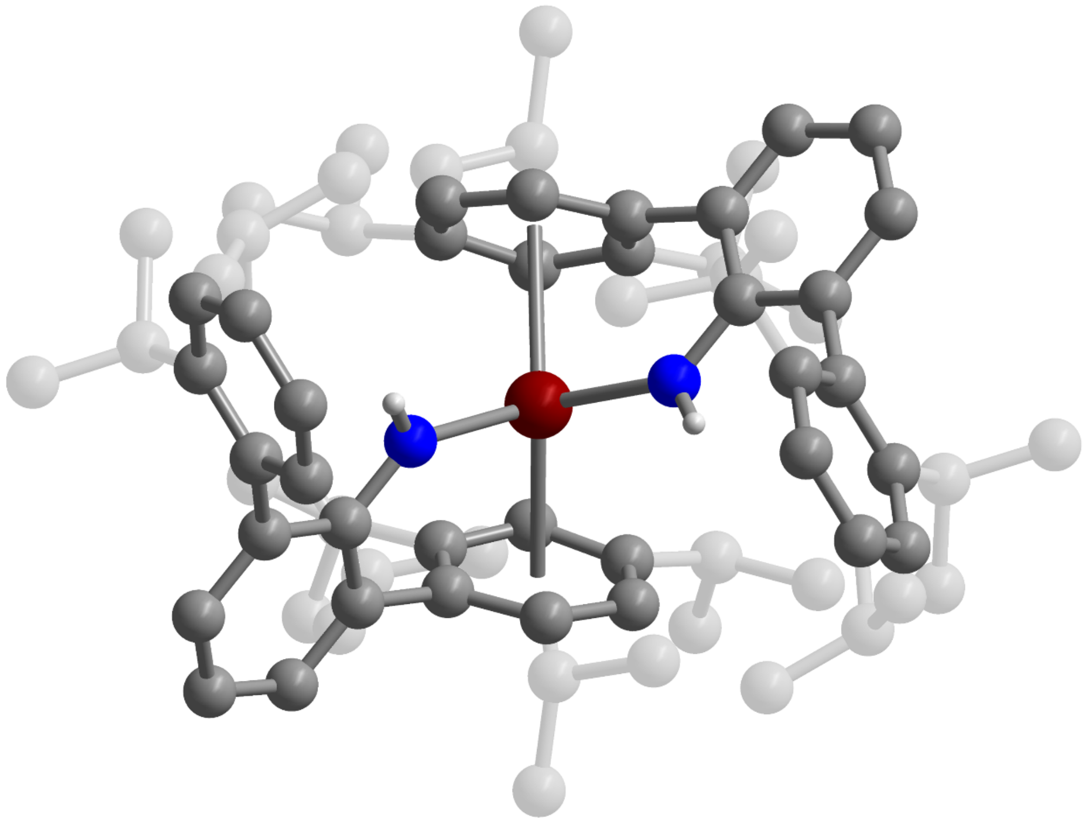

Figure S4. Structure of $(NHAr^*)_2Tb$, **2**, in a crystal of $(NHAr^*)_2Tb\cdot(OC_4H_8)$, where Ar* = 2,6-$(Ar')_2C_6H_3$, Ar' = 2,4,6-$(^iPr)_3C_6H_2$. Dark red, blue, gray, and white-gray spheres represent Tb, N, C, and H atoms, respectively. H atoms bound to all carbon atoms and solvent molecules in the crystal lattice have been omitted for clarity. The isopropyl substituents of the NHAr* ligands have been faded for clarity.

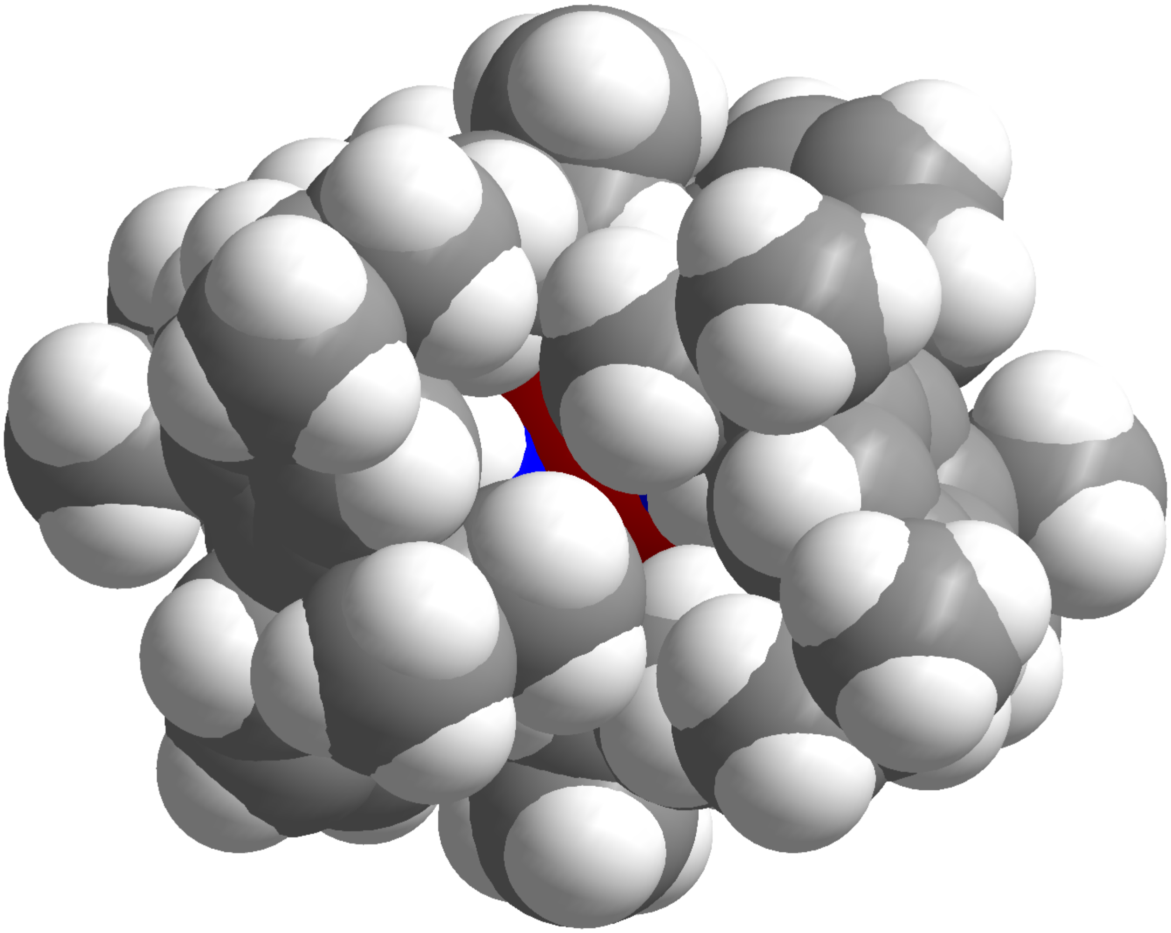

Figure S5. Space filling model of $(NHAr^*)_2Tb$, **2**, in a crystal of $(NHAr^*)_2Tb\cdot(OC_4H_8)$, where Ar* = 2,6-$(Ar')_2C_6H_3$, Ar' = 2,4,6-$(^iPr)_3C_6H_2$. Dark red, blue, gray, and white-gray spheres represent Tb, N, C, and H atoms, respectively. Solvent molecules in the crystal lattice have been omitted for clarity.

2 UV-vis Spectroscopy

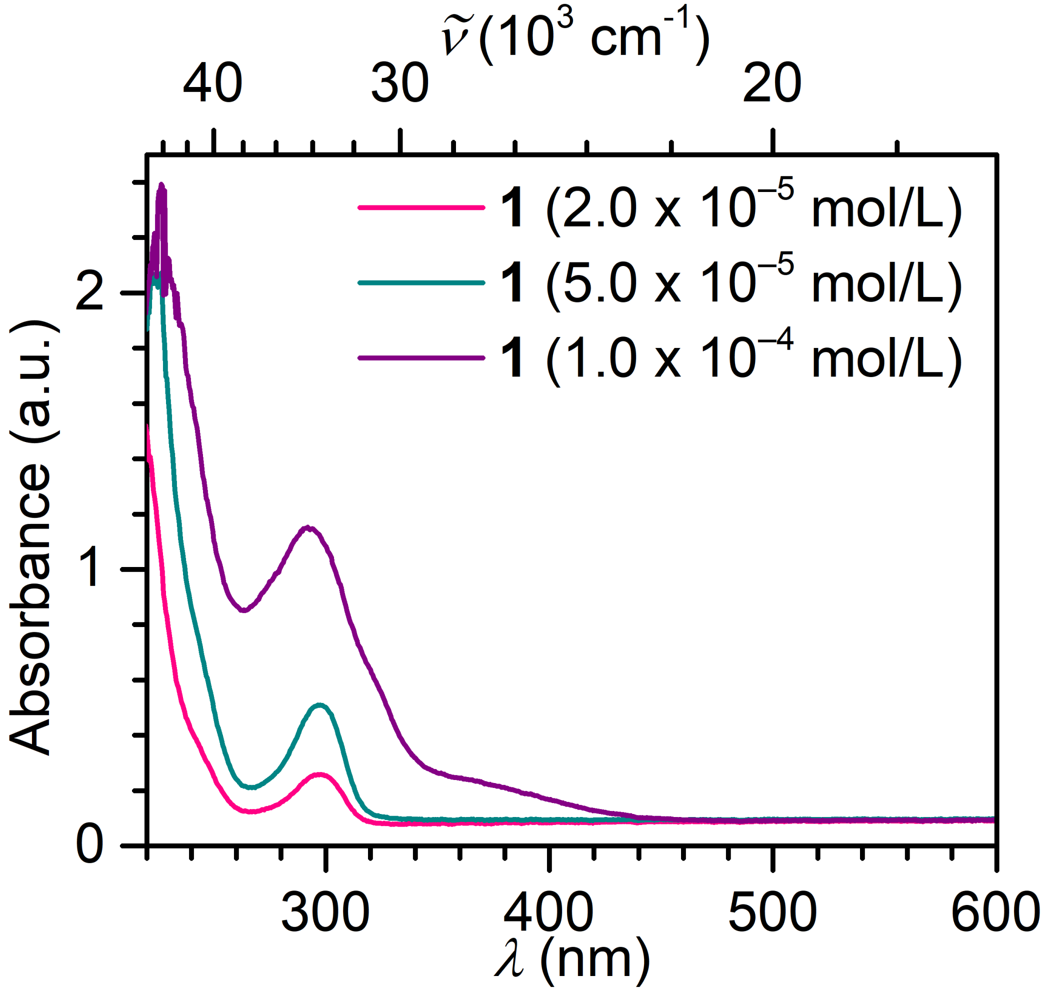

Figure S6. UV-vis absorption spectra of $(NHAr^*)_2TbCl$, **1**, recorded at 100 μmol/L, 50 μmol/L, and 20 μmol/L concentrations in diethyl ether at room temperature.

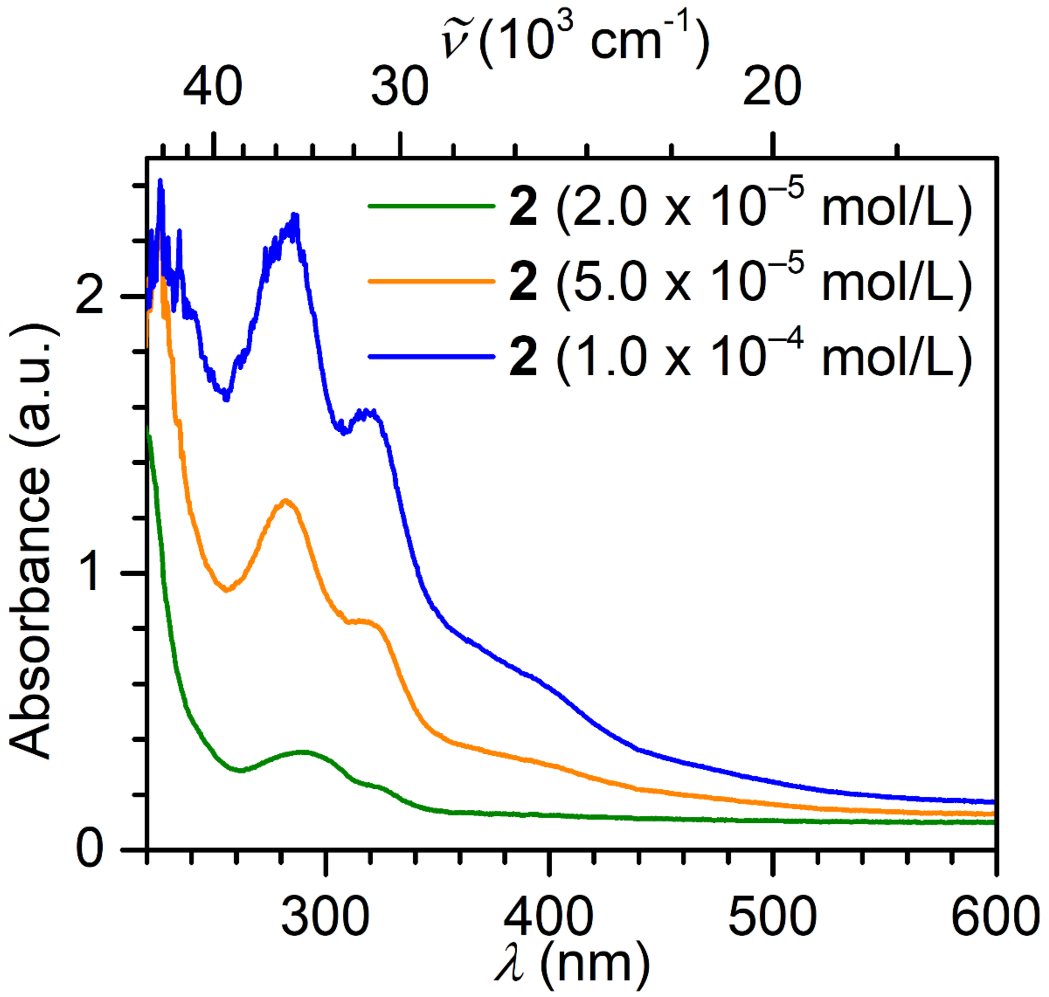

Figure S7. UV-vis absorption spectra of $(NHAr^*)_2Tb$, **2**, recorded at 100 μmol/L, 50 μmol/L, and 20 μmol/L concentrations in diethyl ether at room temperature.

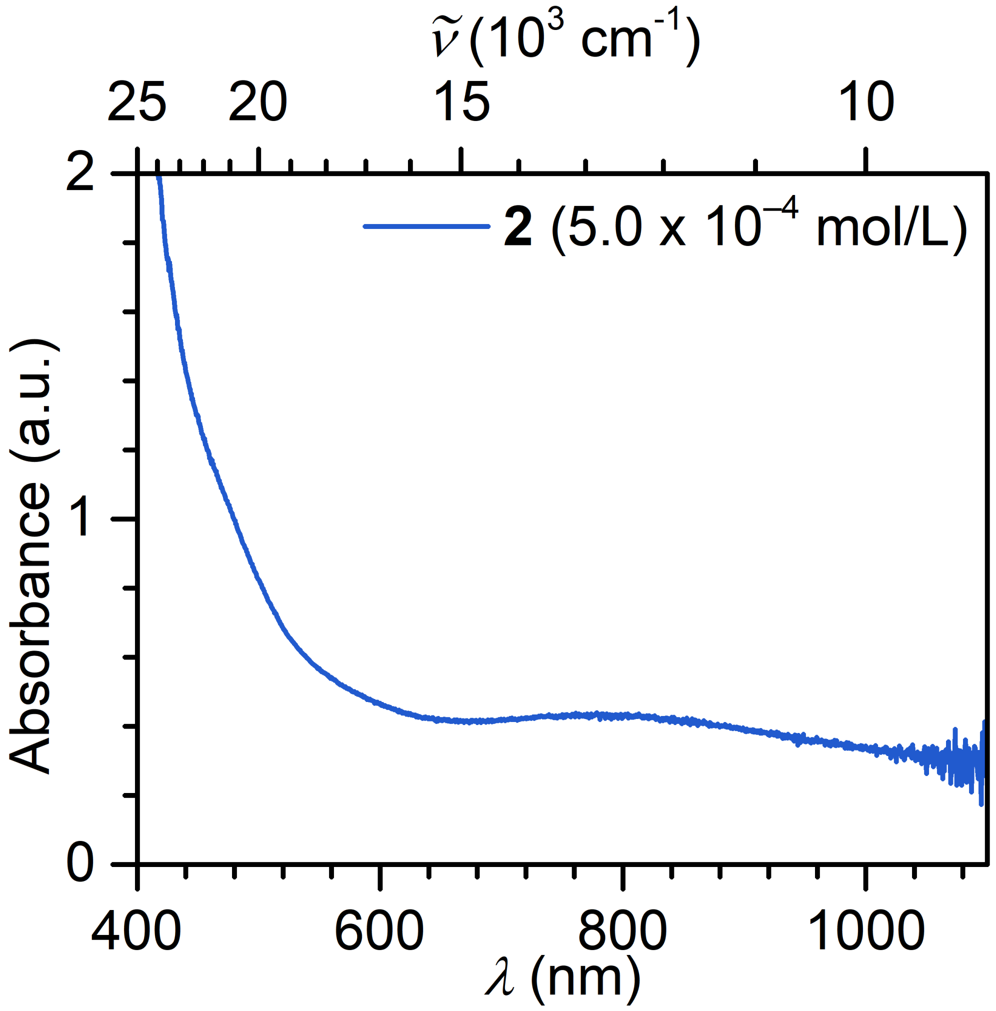

Figure S8. UV-vis absorption spectrum of $(NHAr^*)_2Tb$, **2**, recorded at 500 µmol/L in diethyl ether at room temperature.

3 IR Spectroscopy

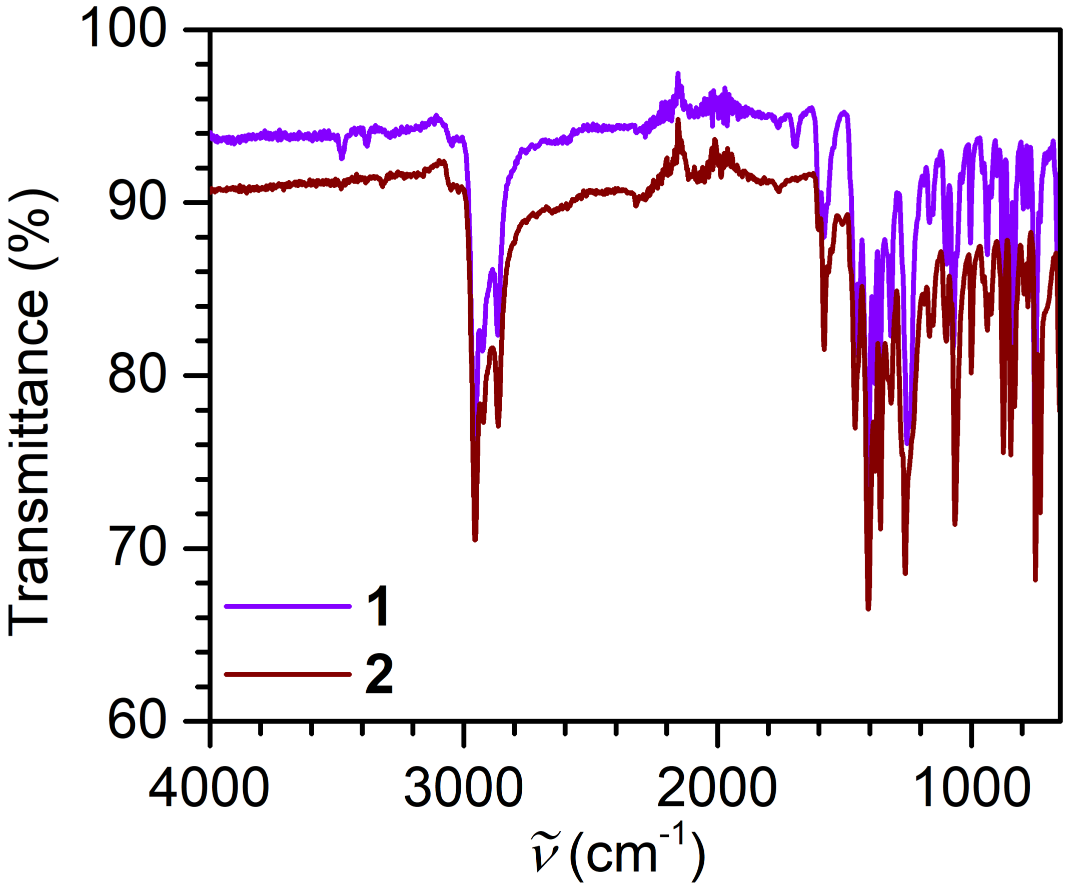

Figure S9. FTIR spectra of $(NHAr^*)_2TbCl$, **1**, and $(NHAr^*)_2Tb$, **2**, collected on crushed crystalline solids under a nitrogen atmosphere.

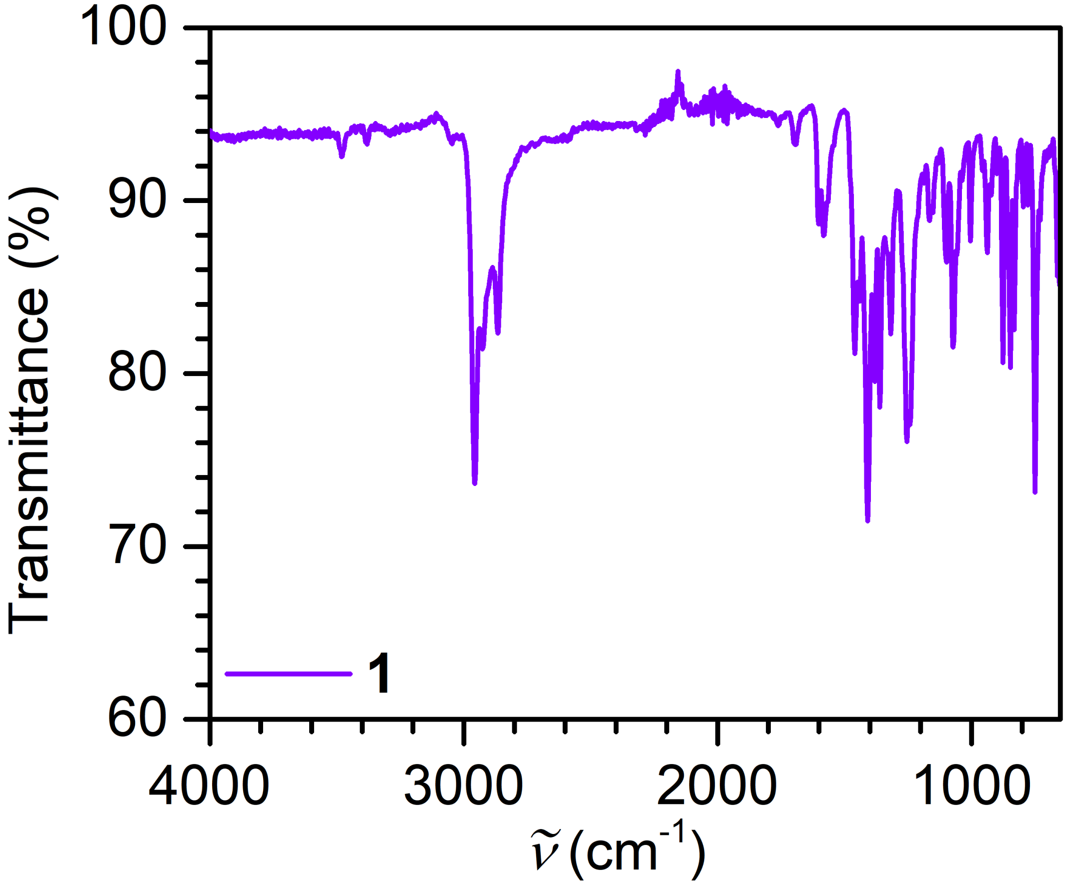

Figure S10. FTIR spectrum of $(NHAr^*)_2TbCl$, **1**, collected on crushed crystalline solids under a nitrogen atmosphere.

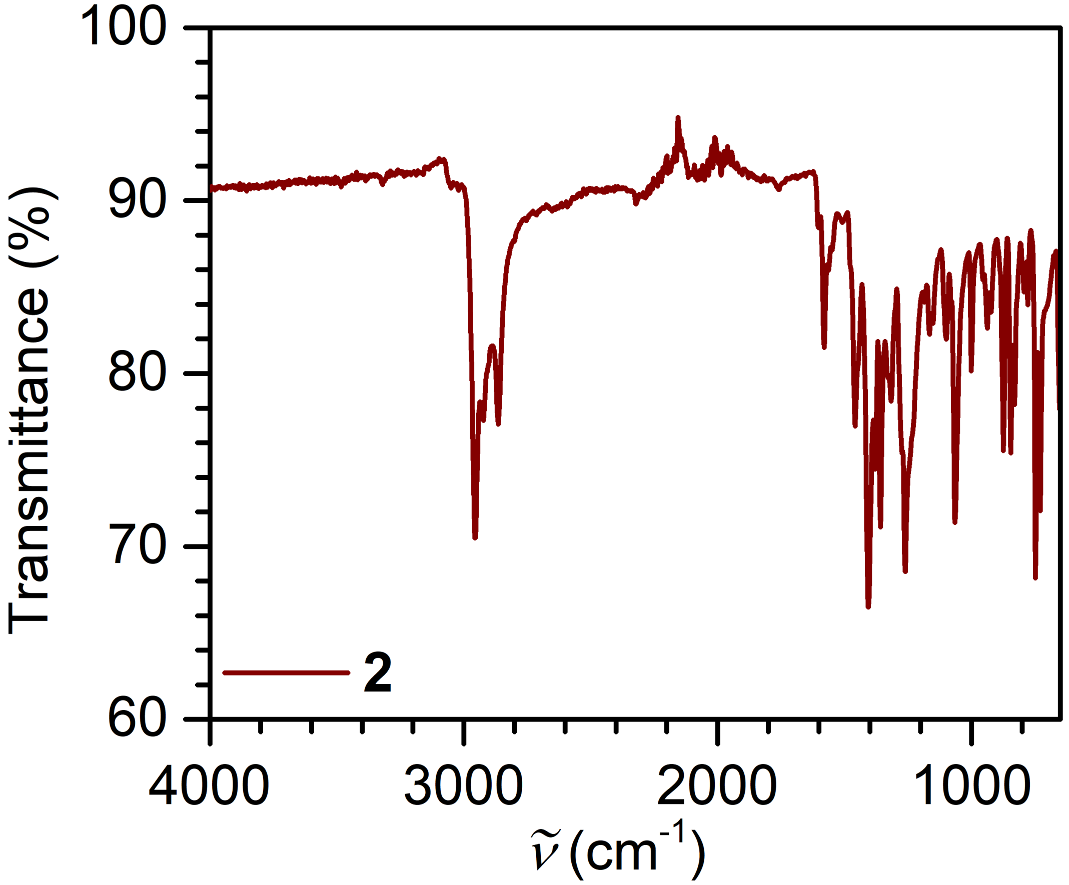

Figure S11. FTIR spectrum of $(NHAr^{*})_2Tb$, **2**, collected on crushed crystalline solids under a nitrogen atmosphere.

4 Magnetic Measurements

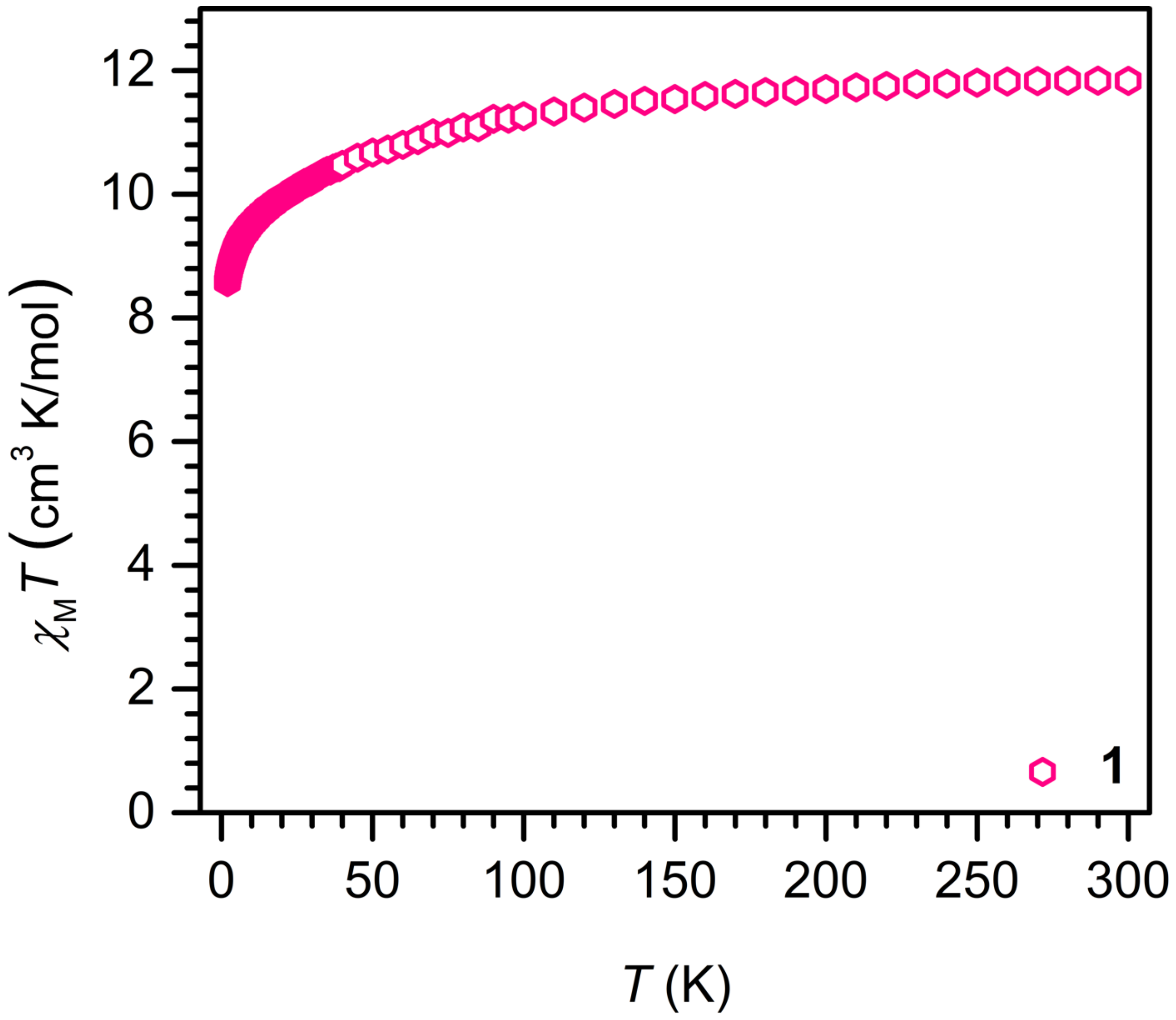

Figure S12. Variable-temperature dc magnetic susceptibility data for a restrained polycrystalline sample of $(NHAr^*)_2TbCl$, **1**, collected under a 0.1 T applied dc field.

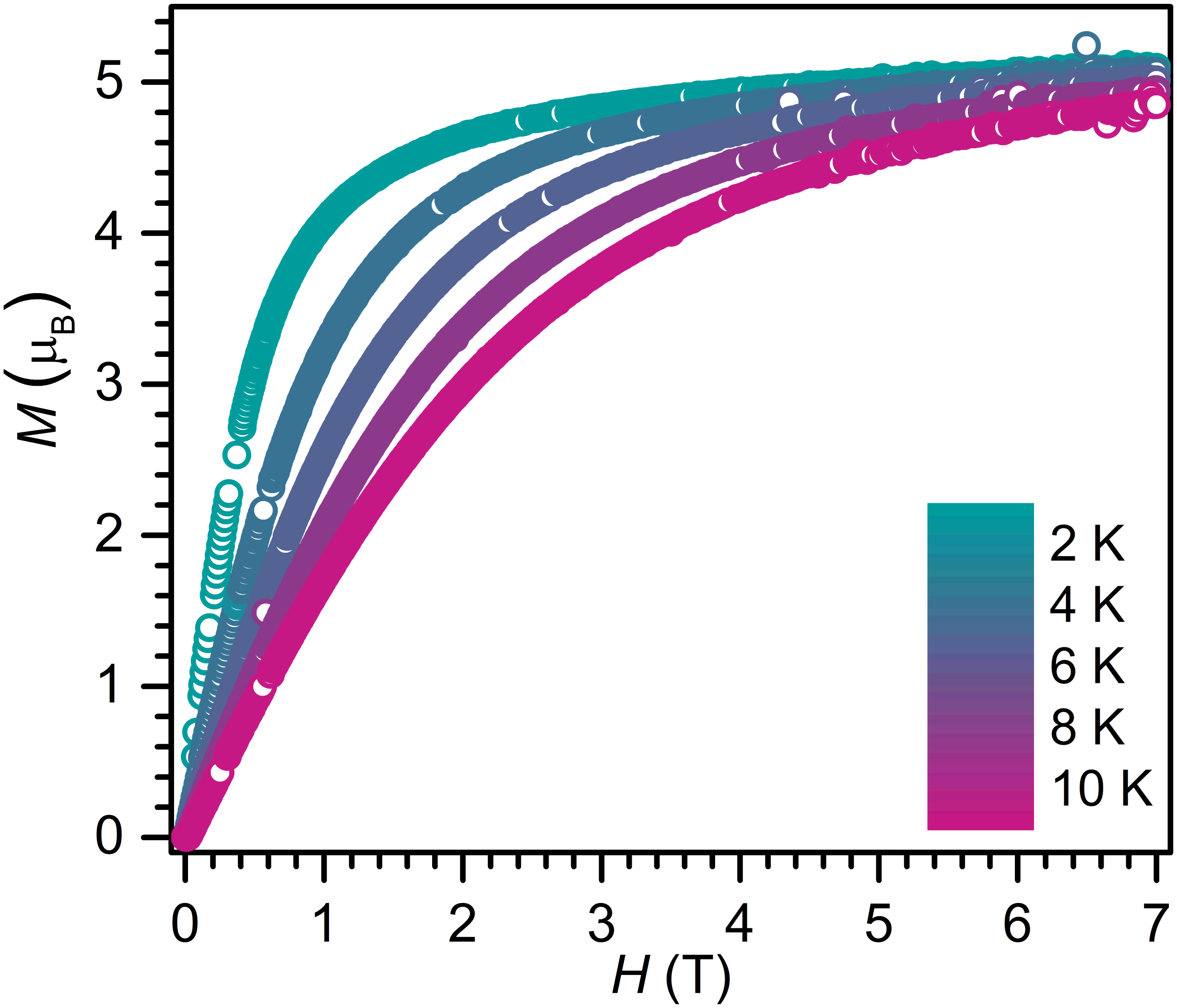

Figure S13. Variable-temperature field-dependent magnetization curves recorded for $(NHAr^*)_2TbCl$, **1**. Measurements were carried out between 0 and 7 T at 2, 4, 6, 8, and 10 K.

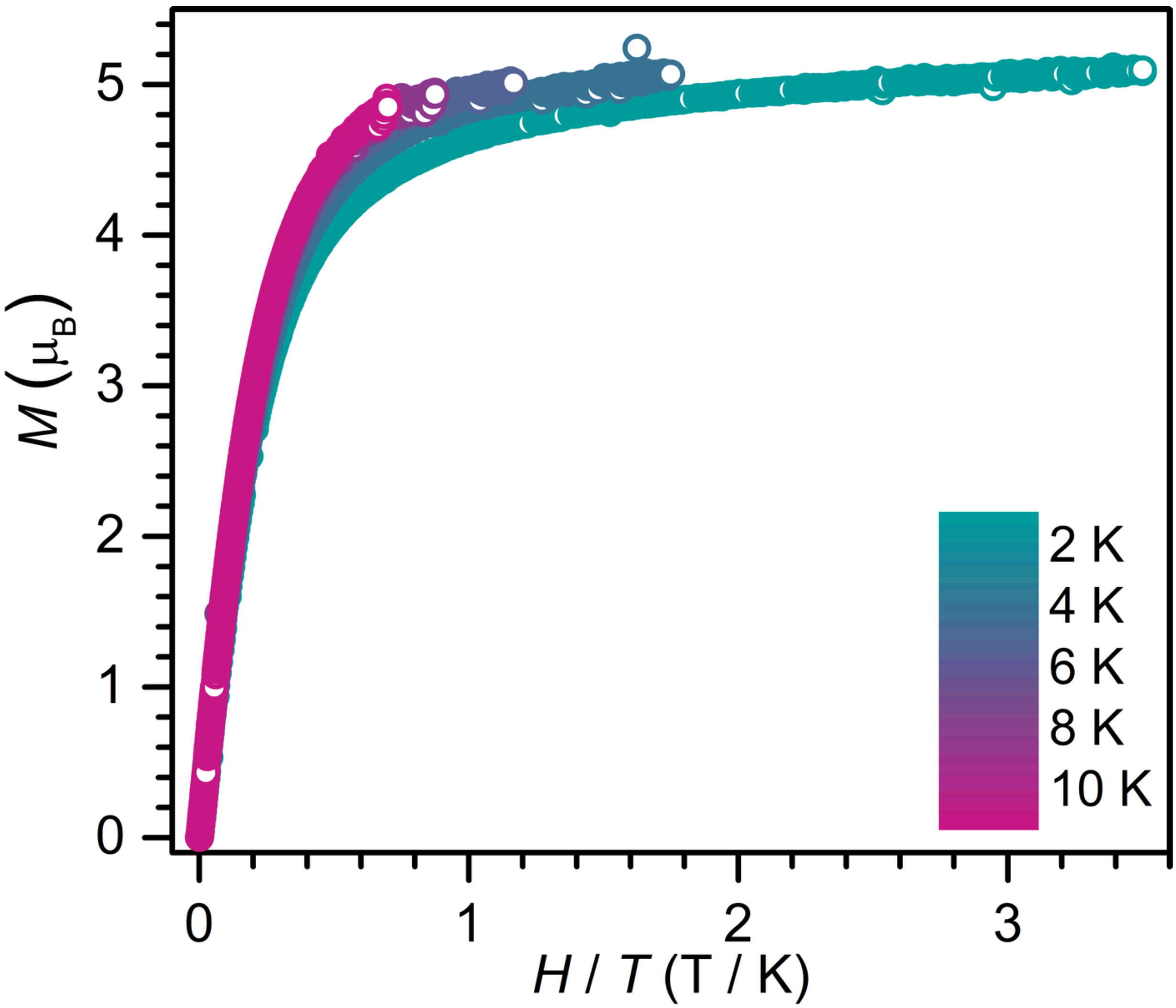

Figure S14. Reduced magnetization curves recorded for $(NHAr^*)_2TbCl$, **1**. Measurements were carried out from 0 to 7 T at 2, 4, 6, 8, and 10 K.

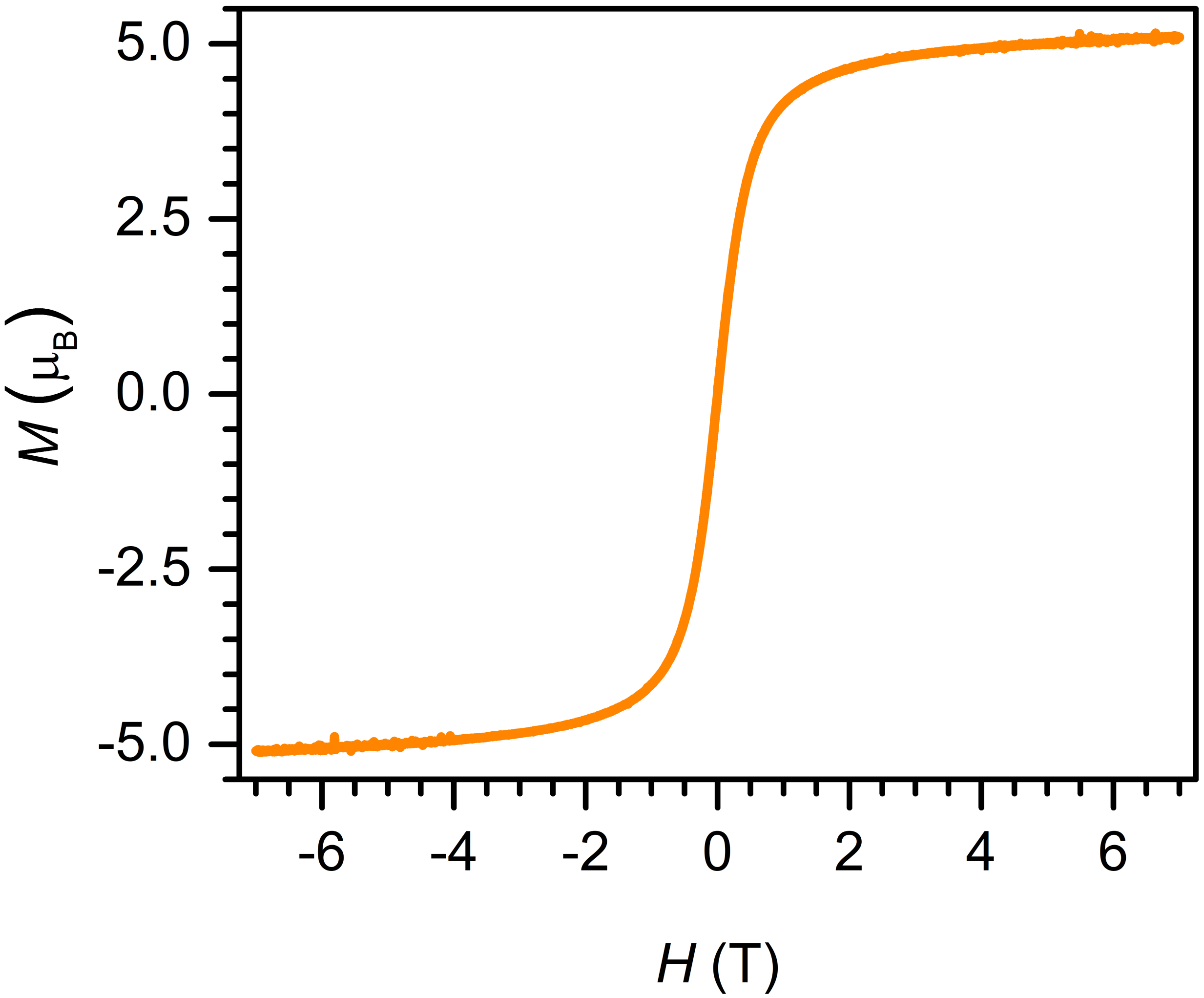

Figure S15. Plot of magnetization (*M*) vs dc magnetic field (*H*) at an average sweep rate of 100 Oe/s for $(NHAr^*)_2TbCl$, **1**, at 1.8 K.

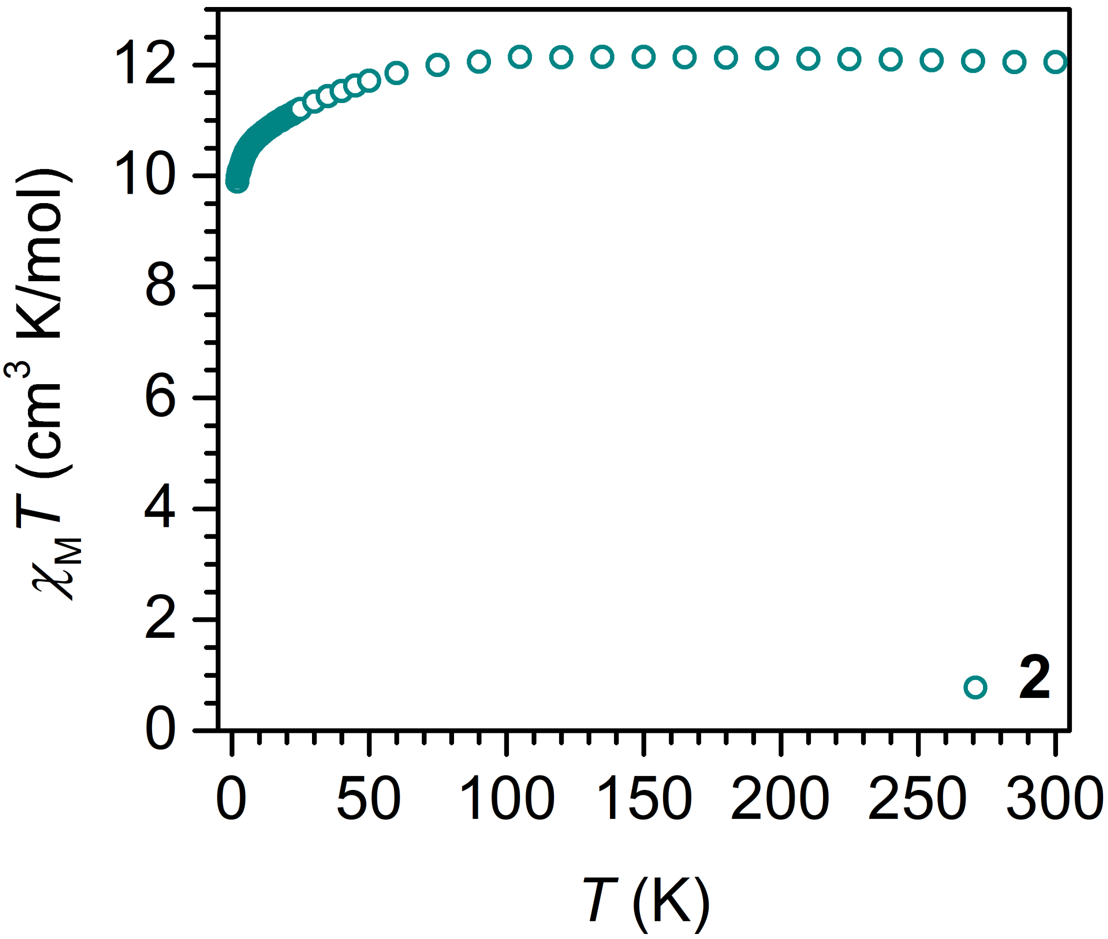

Figure S16. Variable-temperature dc magnetic susceptibility data for a restrained polycrystalline sample of $(NHAr^*)_2Tb$, **2**, collected under a 0.1 T applied dc field.

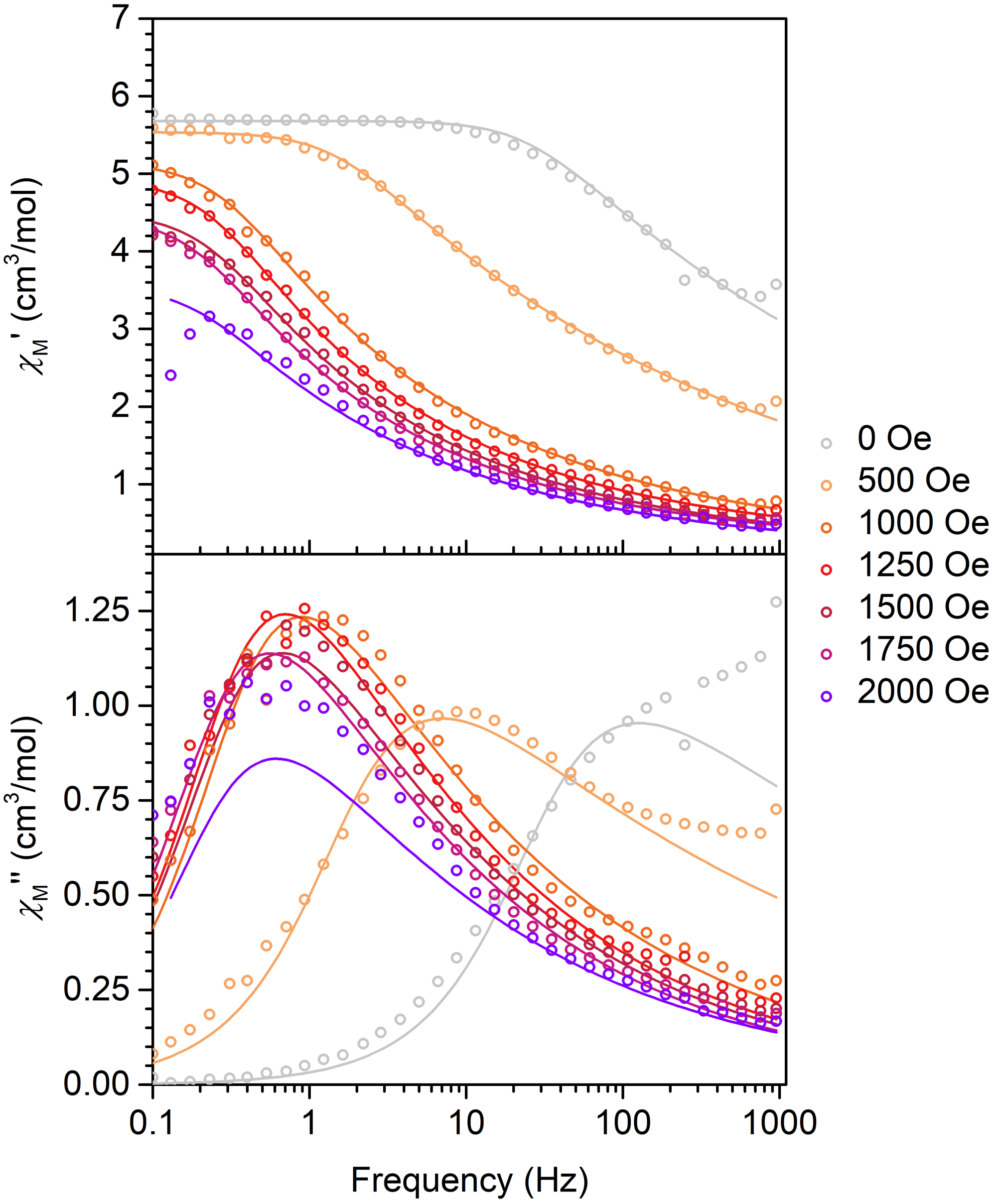

Figure S17. In-phase (χ_M') and out-of-phase (χ_M'') components of the ac magnetic susceptibility for $(NHAr^*)_2Tb$, **2**, at 1.8 K under dc fields ranging from 0 Oe to 2000 Oe. Solid lines are fits to a Cole-Davidson model.

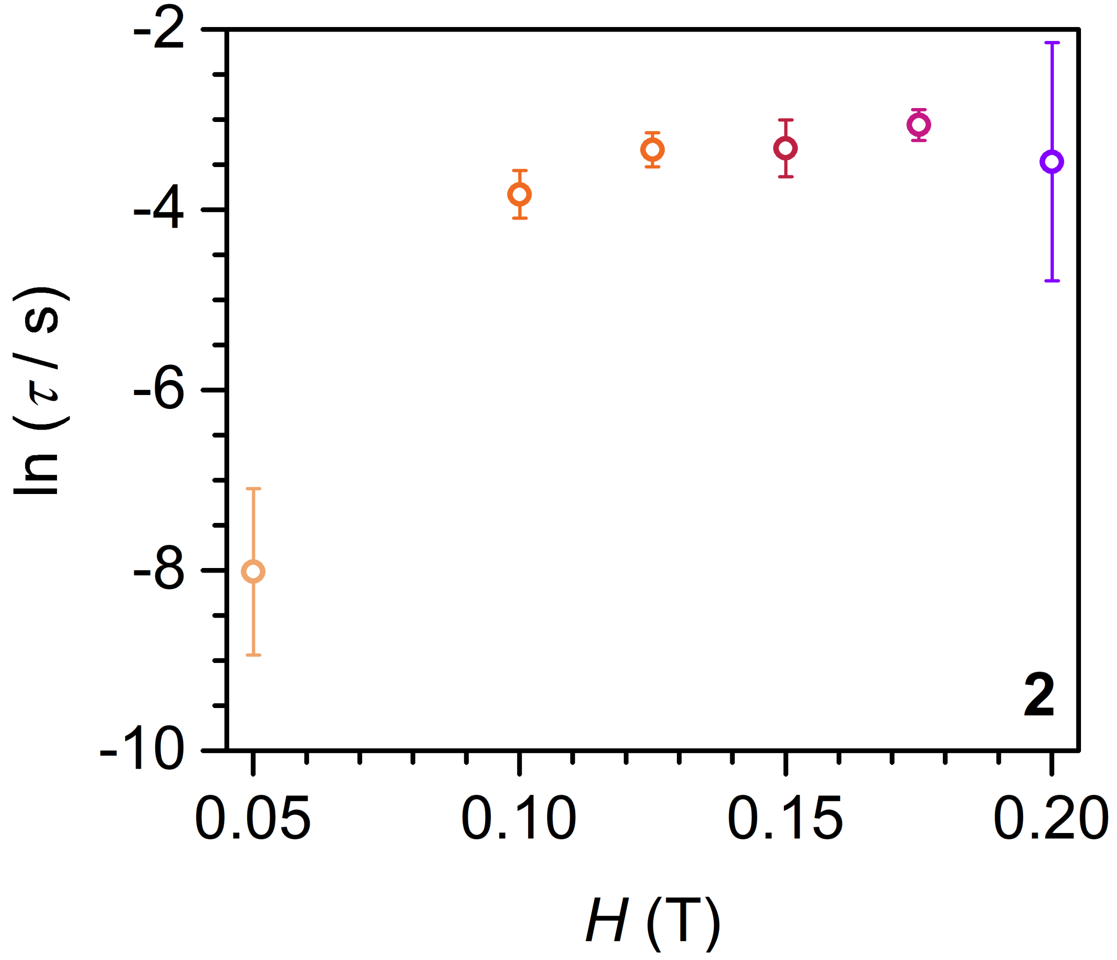

Figure S18. Plot of the natural log of the relaxation time, τ, versus the applied magnetic field obtained from ac measurements, for $(NHAr^*)_2Tb$, **2**, at 1.8 K.

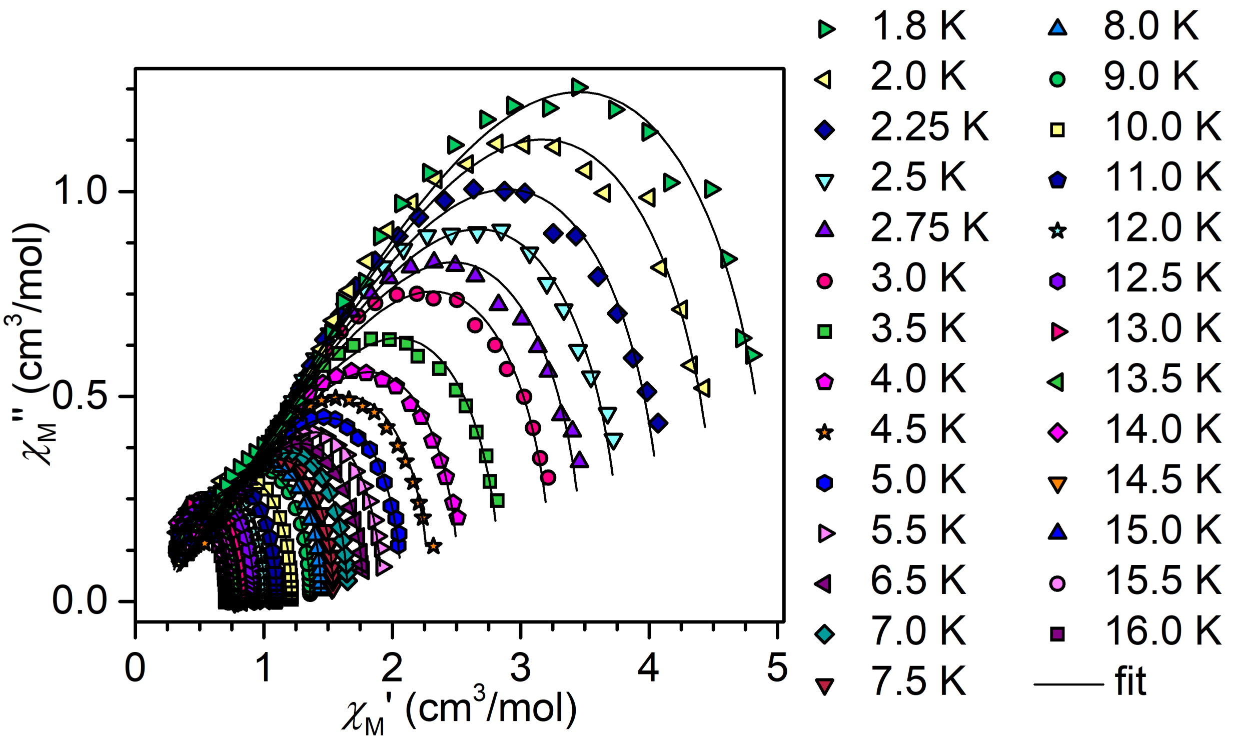

Figure S19. Cole-Cole (Argand) plots for ac susceptibility collected from 1.8 to 16.0 K under 1250 Oe applied dc field for $(NHAr^*)_2Tb$, **2**. Symbols represent the experimental data points and the points representing the fits are connected by black solid lines.

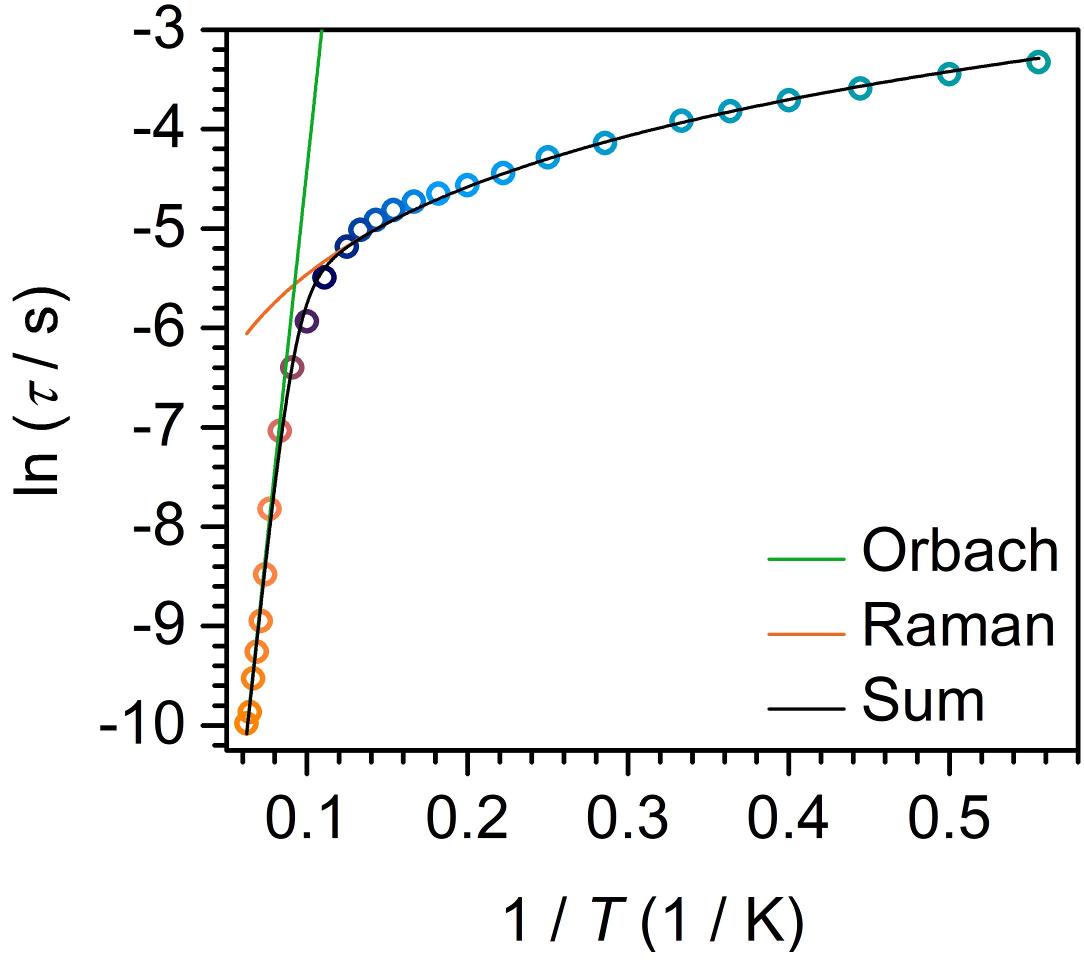

Figure S20. Individual contributions of the multiple magnetic relaxation pathways to the Arrhenius plot of $(NHAr^*)_2Tb$, **2**, at 1250 Oe from 1.8 to 16 K (turquoise to orange circles). Individual parameters used to calculate the contributions are given in Table S2. The black line represents a fit to an Orbach relaxation process and a Raman pathway yielding U_{eff} = 105(3) cm^{-1} and τ_0 = 3.2(7) × 10^{-9} s.

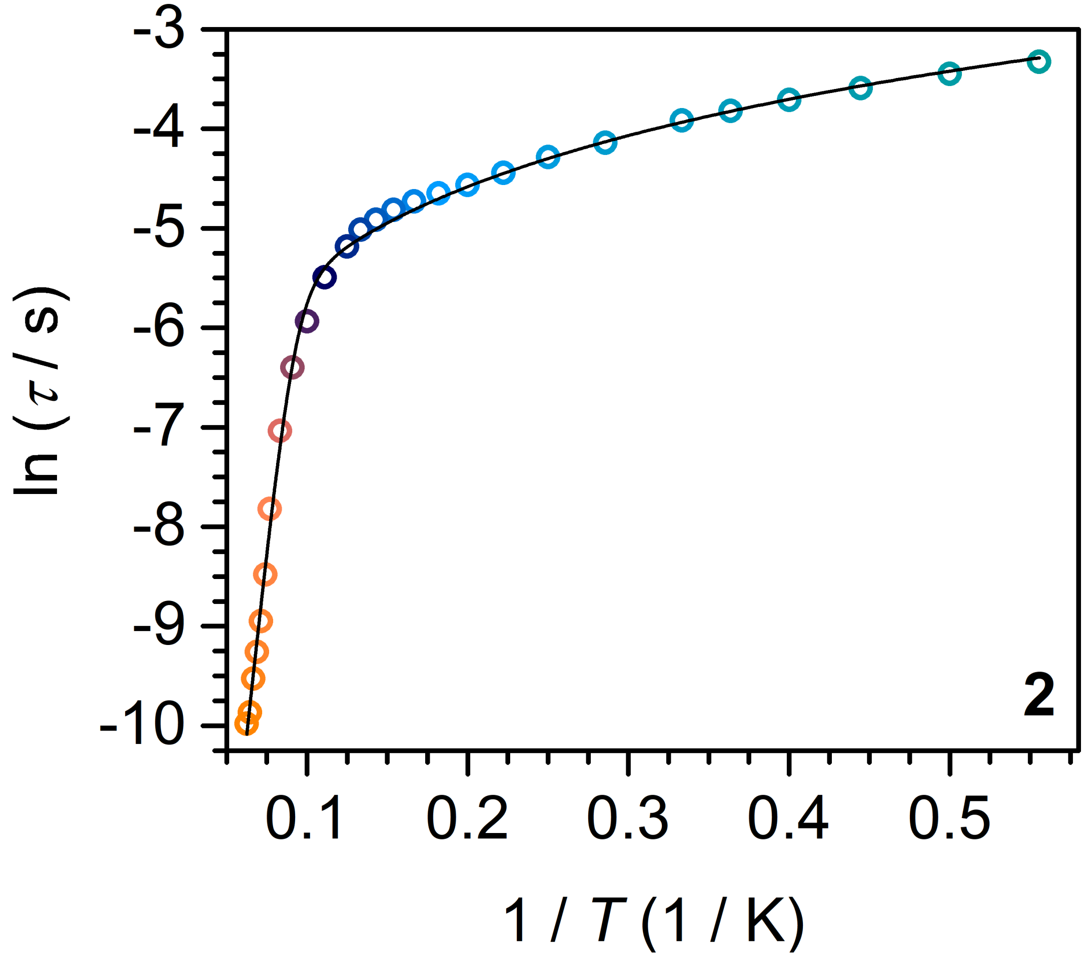

Figure S21. Arrhenius plot of the natural log of the relaxation time, τ, versus the inverse temperature obtained from ac measurements, for $(NHAr^*)_2Tb$, **2**, at 1250 Oe from 1.8 to 16 K (turquoise to orange circles). The black line represents a fit to an Orbach relaxation process and a Raman pathway yielding U_{eff} = 105(3) cm^{-1} and τ_0 = 3.2(7) × 10^{-9} s.

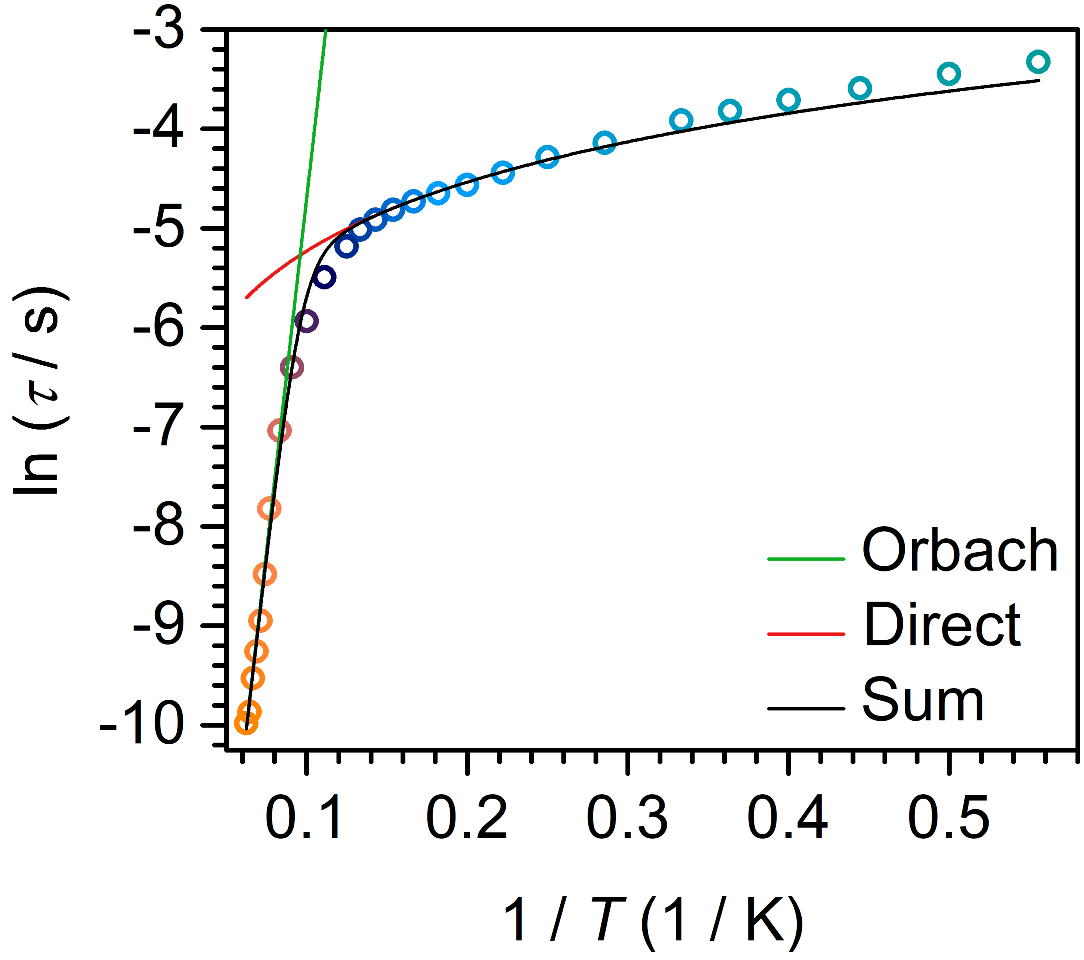

Figure S22. Individual contributions of the multiple magnetic relaxation pathways to the Arrhenius plot of $(NHAr^*)_2Tb$, **2**, at 1250 Oe from 1.8 to 16 K (turquoise to orange circles). Individual parameters used to calculate the contributions are given in Table S2. The black line represents a fit to an Orbach relaxation process and a Direct pathway yielding U_{eff} = 98(4) cm^{-1} and τ_0 = 6.9(3) × 10^{-9} s.

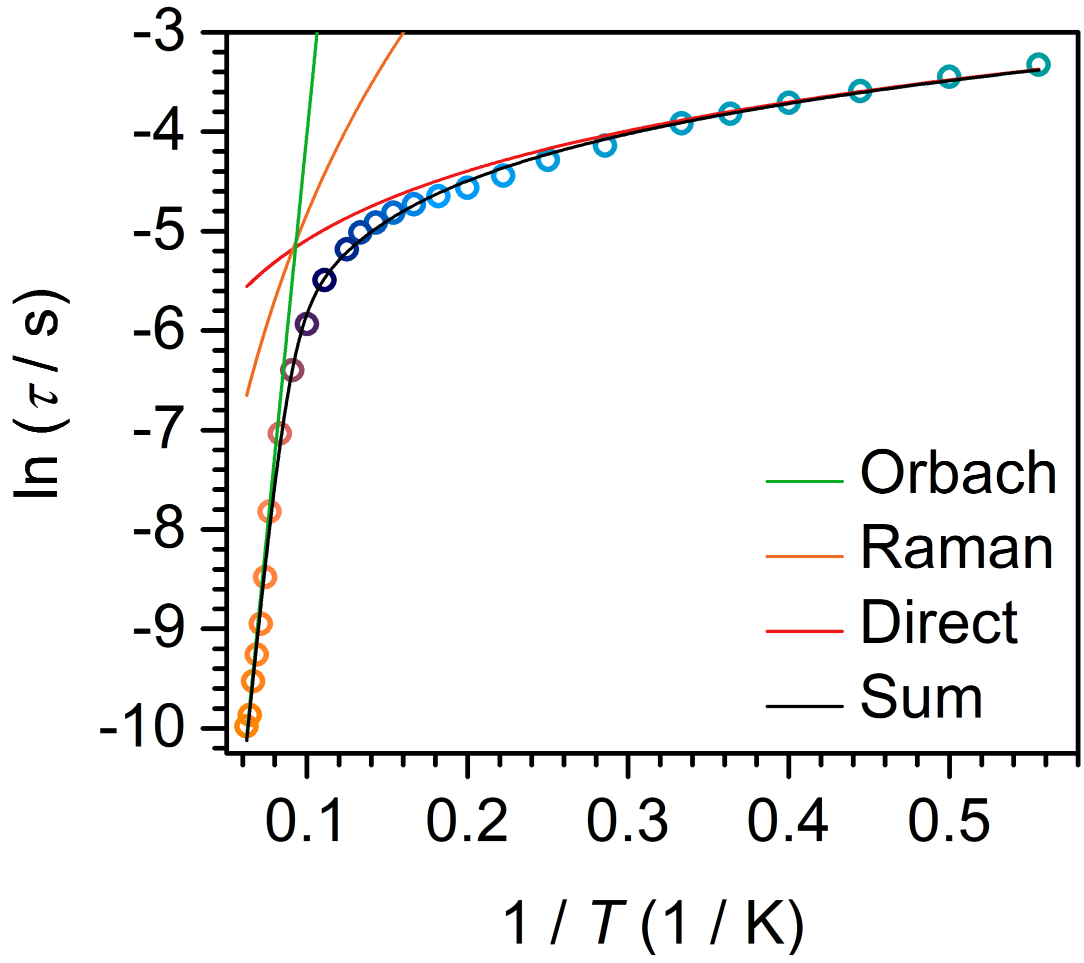

Figure S23. Individual contributions of the multiple magnetic relaxation pathways to the Arrhenius plot of $(NHAr^*)_2Tb$, **2**, at 1250 Oe from 1.8 to 16 K (turquoise to orange circles). Individual parameters used to calculate the contributions are given in Table S2. The black line represents a fit to an Orbach relaxation process, a Raman, and a Direct pathway yielding U_{eff} = 112(5) cm^{-1} and τ_0 = 1.7(7) × 10^{-9} s.

Table S2. Best-fit parameters for the relaxation times of **2** considering different relaxation processes.

Model	A (×10^1 $\mathrm{s^{-1}K^{-1}}$)	C ($\mathrm{s^{-1}K^{-n}}$)	n	τ_0 (×10^{-9} s)	U_{eff} (cm^{-1})	Figure
Orbach+Raman	-	1.2(1)×10^1	1.27(5)	3.2(7)	105(3)	S20
Orbach+Direct	1.8(8)	-	-	6.9(3)	98(4)	S22
Orbach+Raman +Direct	1.6(7)	5(5)×10^{-2}	4(1)	1.7(7)	112(5)	S23

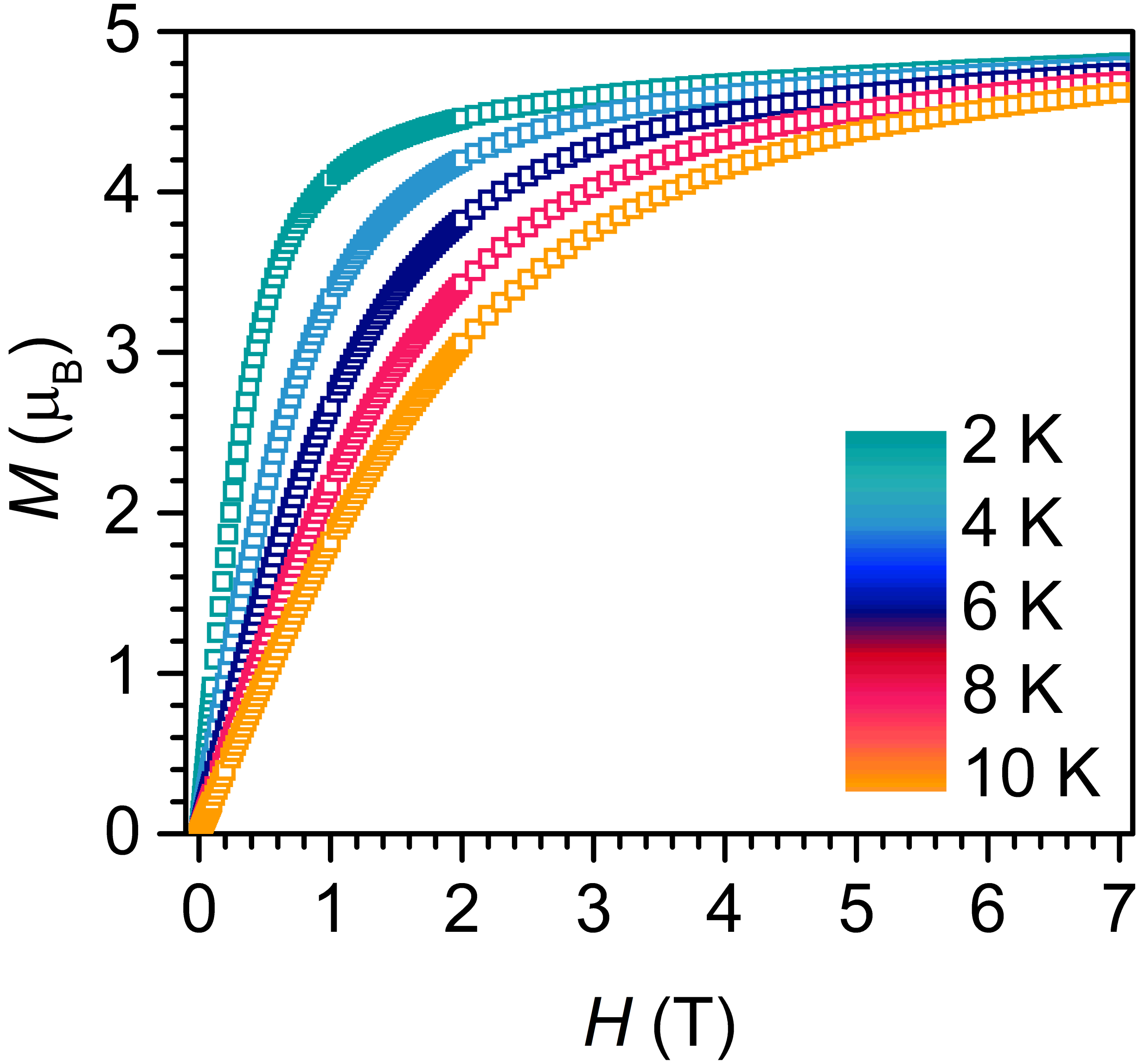

Figure S24. Variable-temperature field-dependent magnetization curves recorded for $(NHAr^*)_2Tb$, **2**. Measurements were carried out from 0 to 7 T at 2, 4, 6, 8, and 10 K.

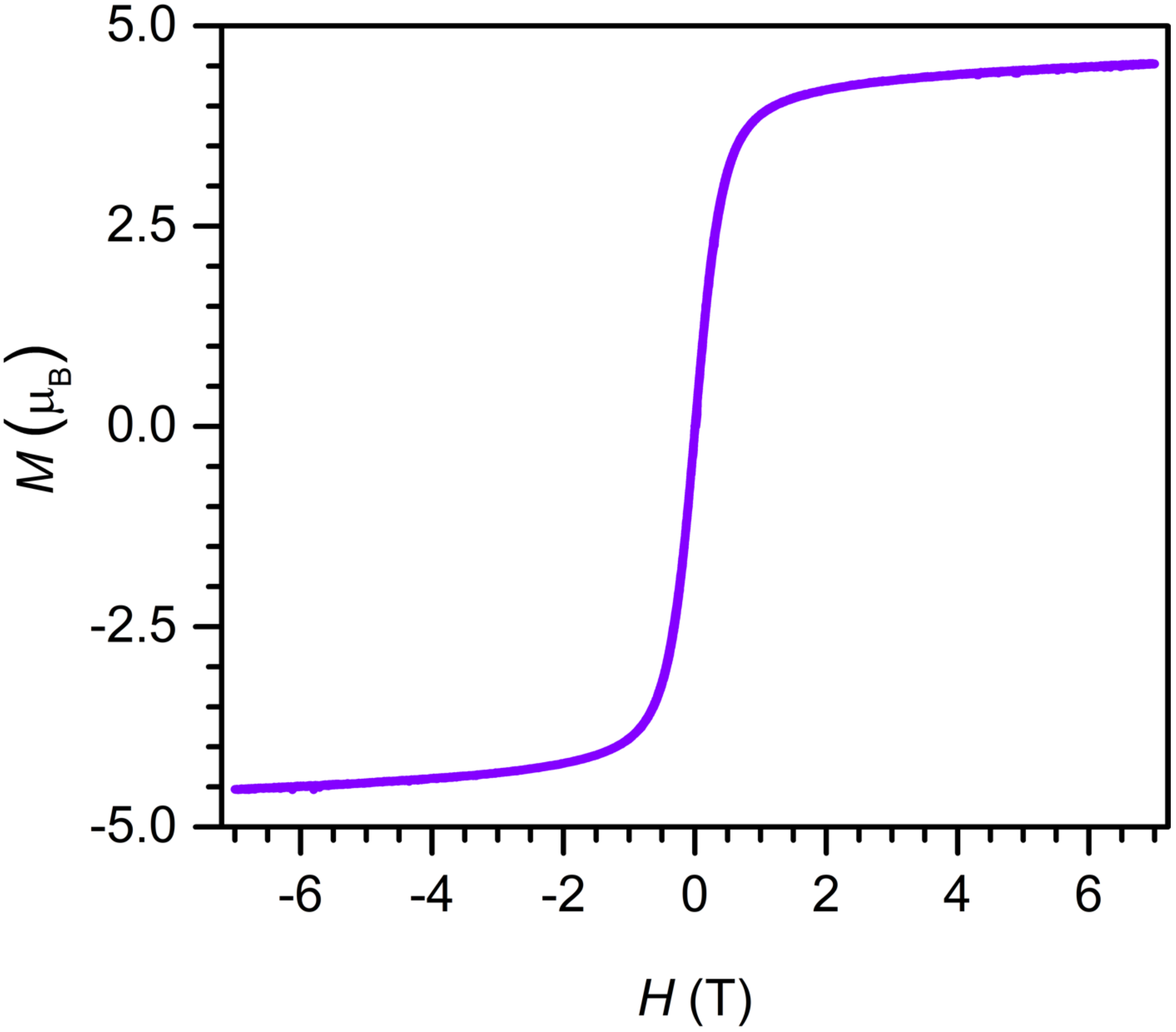

Figure S25. Plot of magnetization (*M*) vs dc magnetic field (*H*) at an average sweep rate of 100 Oe/s for $(NHAr^*)_2Tb$, **2**, at 1.8 K.

5 *Ab Initio* Calculations

To determine the appropriate active space, a set of test calculations were performed taking into account different metal- and ligand-based orbitals. First, as a reference, a CAS(9,7) calculation was carried out on 21 S = 5/2 roots, assuming that the electron introduced upon reduction of **1** populates the 4*f* orbitals. The obtained low-lying energy spectrum is vastly different to the ones obtained from all subsequent calculations and yields an energetically unfavorable solution (> 40k cm^{-1} above below-described CAS(9,15) solution).

Second, a CAS(9,12) was explored, comprising the Tb-based 4*f* and 5*d* orbitals. Upon orbital optimization, the initial atomic Tb 5*d* orbitals hybridize considerably with the antibonding π* orbitals of the coordinating NHAr* ligands. Inspection of the resulting state-averaged compositions reveals a set of 7 lower-lying states below 1659 cm^{-1} that comprise configurations dominantly involving the 5*d*/π* hybrid 1 (herein labelled π^*_1).

A second set of 7 states is found between 6115-7697 cm^{-1} composed of configurations with 5*d*/π* hybrid 2 (π^*_2). The last seven states are located between 11364-12650 cm^{-1} and bear 5*d*/π* configurations with hybrid orbital 3 (π^*_3). Notably, the three populated hybrid orbitals resemble very closely the corresponding lowest-lying ligand-based π* orbitals that are obtained in a test calculation in the absence of the Tb ion on the $[(NHAr^*)_2]^{2-}$ moiety. These orbitals are labelled π^*_1-π^*_3 in Table S3.

Since we deployed the fragment guess method (merging metal-only and ligand-only orbitals to attain a perfectly ionic starting orbital set), we included the orbitals in question in the active space alongside the Tb 5*d* orbitals to assess the necessity of the inclusion of ligand-based orbitals (CAS(9,15)). Indeed, we found that the same three orbitals share a 1 e^- occupancy (~0.33 per orbital) while the Tb 5*d* orbitals slightly gain ligand contributions but remain essentially unoccupied. The inclusion of these orbitals has profound effects on the low-lying energy manifold, most notably reducing the overall splitting by ~23%, likely due to improved compensation for dynamic electron correlation.

Subsequently, we attempted the inclusion of further virtual Tb orbitals via the 6*s* orbital in a (CAS(9,16)). Here, the 6*s* orbital hybridized substantially with the ligand orbitals, to the extent that only ~30% contribution stems from the initial 6*s* orbital (judged via Loewdin population analysis) while being essentially unoccupied. Although carried out from scratch, this calculation yielded three hybrid orbitals in accordance with the CAS(9,15) calculated π^*_1-π^*_3 orbitals, while leaving the low-lying energy spectrum essentially unchanged. Consequently, we ruled out the involvement of any significant Tb 6*s* contribution to the electronic structure of **2** (Table S4).

Lastly, we attempted the inclusion of further low-lying ligand-based π* orbitals. Here, the lowest-lying π^*_4 (not shown) orbital obtained from the above CAS(9,15) was rotated into the active space and orbital optimization was conducted. However, although close, convergence could not be achieved over several attempts due to oscillating behavior in the orbital gradient. The semi-converged low-lying energy spectrum is provided in Table S5 for comparative purposes only.

Table S3. State-average occupation numbers of the optimized active orbitals in different CAS calculations in the S = 7/2 state of **2** over 21 roots. The occupation of each $4f$ orbital was omitted since a value of 1.143 was found in each case, in accordance with a $4f^8$ occupation.

Active Space	*State-average occupation numbers*									
	π^*_1	π^*_2	π^*_3	$5d_1$	$5d_2$	$5d_3$	$5d_4$	$5d_5$	$6s$	π^*_4
CAS(9,12)	0.333	0.333	0.316	0.019	0.001	-	-	-	-	-
CAS(9,15)	0.334	0.334	0.333	0.002	0.002	0.002	0.002	0.002	-	-
*CAS(9,16)	0.334	0.334	0.333	0.002	0.002	0.002	0.002	0.002	-	0.001
‡CAS(9,16)	0.334	0.334	0.333	0.002	0.002	0.002	0.002	0.002	0.000	-

*7 $4f$,5 $5d$, 4 π^*. ‡7 $4f$, 5 $5d$,1 $6s$, 3 π^*.

Table S4. Single point energies (H) for each converged active space test calculation.

CAS(9,7)	-14598.145471651680
CAS(9,12)	-14598.318455464001
CAS(9,15)	-14598.332389546304
*CAS(9,16)	-14598.332885297743
‡CAS(9,16)	-14598.332428416186

*7 $4f$,5 $5d$, 4 π^*. ‡7 $4f$, 5 $5d$,1 $6s$, 3 π^*.

Table S5. CASSCF state-averaged energies for the 21 S = 7/2 (S = 5/2 for CAS(9,7)) states in cm^{-1} across different active spaces.

State no.	CAS(9,7)	CAS(9,12)	CAS(9,15)	CAS(9,16)*	CAS(9,16)‡
1	0.0	0.0	0.0	0.0	0.0
2	96.6	132.2	158.6	179.8	158.1
3	120.4	445.9	435.9	391.4	435.6
4	290.4	700.7	569.6	461.8	568.7
5	438.1	795.0	687.8	714.0	686.4
6	567.1	1569.0	1385.4	1389.5	1385.0
7	606.8	1658.7	1494.4	1420.0	1488.9
8	633.9	6114.8	6595.5	6560.2	6592.1
9	658.5	6293.7	6842.9	6810.4	6839.5
10	1084.7	6561.5	6955.2	6912.4	6950.0
11	1094.9	6701.6	7128.1	7003.7	7122.8
12	7028.6	6941.7	7286.5	7094.3	7281.3
13	7138.0	7568.3	7885.1	7867.3	7881.1
14	7215.6	7696.8	8019.7	7920.6	8010.2
15	7227.5	11363.9	9470.8	9471.8	9471.2
16	7355.8	11576.6	9522.8	9546.8	9522.6
17	7401.7	12189.2	9621.2	9648.0	9622.8
18	7443.3	12248.7	9840.5	9730.9	9840.5
19	31431.5	12441.7	9849.2	9881.4	9850.5
20	31813.4	12645.7	10193.9	10180.8	10194.4
21	32349.0	12650.5	10278.2	10229.4	10274.8

*7 4*f*, 5 5*d*, 4 π*, not converged. ‡7 4*f*, 5 5*d*,1 6*s*, 3 π*.

Table S6. Relative CASSCF state-averaged energies for the 21 $S = 7/2$ and 21 $S = 5/2$ states in cm^{-1} with TZVP and QZVP basis set for Tb.

Tb basis set	TZVP		QZVP	
Spin	$S = 7/2$	$S = 5/2$	$S = 7/2$	$S = 5/2$
1	0.0	0.0	0.0	0.0
2	153.8	79.0	155.1	72.4
3	384.6	107.5	362.5	113.8
4	545.3	329.2	531.1	307.3
5	627.4	438.9	596.7	434.0
6	1305.0	936.0	1288.9	927.5
7	1422.0	1221.3	1402.4	1208.5
8	6557.1	5807.4	6533.6	5785.3
9	6795.5	5964.9	6773.2	5937.7
10	6908.1	6065.4	6863.0	6023.6
11	7101.9	6315.7	7045.6	6280.1
12	7225.0	6603.8	7191.2	6553.3
13	7813.9	7007.4	7777.6	6964.4
14	7951.1	7362.2	7910.3	7322.9
15	9323.7	8296.6	9340.0	8305.5
16	9418.0	8543.9	9428.5	8549.9
17	9519.5	8720.4	9511.5	8713.8
18	9726.0	8836.4	9723.2	8814.3
19	9748.2	8983.1	9733.2	8970.0
20	10103.1	9200.7	10095.6	9183.6
21	10210.1	9254.0	10198.1	9236.9
Absolute difference between State 21 ($S = 7/2$) and State 1 ($S = 5/2$) manifold (cm^{-1})	-9210.0[a]		-9193.4[a]	

[a]The energy of the highest $S = 7/2$ and lowest $S = 5/2$ states overlap considerably as indicated by the negative value.

Table S7. Relative energies of the 50 lowest lying CASSCF(9,15)/NEVPT2/QDPT states, which were obtained considering 21 S = 7/2 and 21 S = 5/2 states.

1	0.0	26	1572.1
2	0.0	27	2026.4
3	160.8	28	2026.4
4	160.8	29	2069.5
5	290.8	30	2069.5
6	290.8	31	2156.8
7	365.0	32	2156.8
8	365.0	33	2285.9
9	483.6	34	2285.9
10	483.6	35	2394.6
11	577.7	36	2394.6
12	577.7	37	2429.7
13	616.3	38	2429.7
14	616.3	39	2457.9
15	726.5	40	2457.9
16	726.5	41	2595.6
17	753.7	42	2595.6
18	753.7	43	2703.7
19	834.1	44	2703.7
20	834.1	45	2803.4
21	1016.8	46	2803.4
22	1016.8	47	2964.4
23	1254.9	48	2964.4
24	1254.9	49	3644.2
25	1572.1	50	3644.2

6 References

(1) Ryan, A. J.; Darago, L. E.; Balasubramani, S. G.; Chen, G. P.; Ziller, J. W.; Furche, F.; Long, J. R.; Evans, W. J. Synthesis, Structure, and Magnetism of Tris(Amide) $[Ln\{N(SiMe_3)_2\}_3]^{1-}$ Complexes of the Non-Traditional +2 Lanthanide Ions. *Chem. Eur. J.* **2018**, *24* (30), 7702–7709. https://doi.org/10.1002/chem.201800610.

(2) Bates, E.; Ziller, J. W.; Furche, F.; Evans, W. J. Completing the Series of +2 Ions for the Lanthanide Elements: Synthesis of Molecular Complexes of Pr^{2+},Gd^{2+},Tb^{2+}, and Lu^{2+}. *J. Am. Chem. Soc* **2013**, *135* (26), 9857–9868. https://doi.org/10.1021/ja403753j.

(3) Gould, C. A.; McClain, K. R.; Yu, J. M.; Groshens, T. J.; Furche, F.; Harvey, B. G.; Long, J. R. Synthesis and Magnetism of Neutral, Linear Metallocene Complexes of Terbium(II) and Dysprosium(II). *J. Am. Chem. Soc.* **2019**, *141* (33), 12967–12973. https://doi.org/10.1021/jacs.9b05816.

(4) Jin, P. B.; Luo, Q. C.; Gransbury, G. K.; Vitorica-Yrezabal, I. J.; Hajdu, T.; Strashnov, I.; McInnes, E. J. L.; Winpenny, R. E. P.; Chilton, N. F.; Mills, D. P.; Zheng, Y. Z. Thermally Stable Terbium(II) and Dysprosium(II) Bis-Amidinate Complexes. *J. Am. Chem. Soc.* **2023**, *145* (51), 27993–28009. https://doi.org/10.1021/jacs.3c07978.
